# Immersive virtual reality for learning exoskeleton-like virtual walking: a feasibility study

**DOI:** 10.1186/s12984-024-01482-y

**Published:** 2024-11-01

**Authors:** Antonio Rodríguez-Fernández, Alex van den Berg, Salvatore Luca Cucinella, Joan Lobo-Prat, Josep M. Font-Llagunes, Laura Marchal-Crespo

**Affiliations:** 1 Biomechanical Engineering Lab, Department of Mechanical Engineering and Research Center for Biomedical Engineering, Universitat Politècnica de Catalunya, Barcelona, 08028 Spain; 2 Institut de Recerca Sant Joan de Déu, Esplugues de Llobregat, 08950 Spain; 3 Department of Cognitive Robotics, Delft University of Technology, Delft, 2628 The Netherlands; 4 Department of Rehabilitation Medicine, Erasmus University Medical Center, Rotterdam, 3015 The Netherlands; 5 ABLE Human Motion, Barcelona, 08028 Spain

**Keywords:** Augmented feedback, Immersive virtual reality, Head-mounted display, Lower-limb, Motor learning, Person perspective, Spinal cord injury, Visual feedback, Virtual reality, Walking, Wearable exoskeleton

## Abstract

**Purpose:**

Virtual Reality (VR) has proven to be an effective tool for motor (re)learning. Furthermore, with the current commercialization of low-cost head-mounted displays (HMDs), immersive virtual reality (IVR) has become a viable rehabilitation tool. Nonetheless, it is still an open question how immersive virtual environments should be designed to enhance motor learning, especially to support the learning of complex motor tasks. An example of such a complex task is triggering steps while wearing lower-limb exoskeletons as it requires the learning of several sub-tasks, e.g., shifting the weight from one leg to the other, keeping the trunk upright, and initiating steps. This study aims to find the necessary elements in VR to promote motor learning of complex virtual gait tasks.

**Methods:**

In this study, we developed an HMD-IVR-based system for training to control wearable lower-limb exoskeletons for people with sensorimotor disorders. The system simulates a virtual walking task of an avatar resembling the sub-tasks needed to trigger steps with an exoskeleton. We ran an experiment with forty healthy participants to investigate the effects of first- (1PP) vs. third-person perspective (3PP) and the provision (or not) of concurrent visual feedback of participants’ movements on the walking performance – namely number of steps, trunk inclination, and stride length –, as well as the effects on embodiment, usability, cybersickness, and perceived workload.

**Results:**

We found that all participants learned to execute the virtual walking task. However, no clear interaction of perspective and visual feedback improved the learning of all sub-tasks concurrently. Instead, the key seems to lie in selecting the appropriate perspective and visual feedback for each sub-task. Notably, participants embodied the avatar across all training modalities with low cybersickness levels. Still, participants’ cognitive load remained high, leading to marginally acceptable usability scores.

**Conclusions:**

Our findings suggest that to maximize learning, users should train sub-tasks sequentially using the most suitable combination of person’s perspective and visual feedback for each sub-task. This research offers valuable insights for future developments in IVR to support individuals with sensorimotor disorders in improving the learning of walking with wearable exoskeletons

## Introduction

Virtual Reality (VR) has been demonstrated to be a promising tool to support motor (re)learning [1, 2]. Over the years, the use of VR has become increasingly popular in supporting training in a variety of fields such as medicine, rehabilitation, psychology, surgical training, education, industry, sports, and exercise [2–10]. However, the most common displays utilized are standard computer screens, televisions, or wall projection systems (i.e., 2D screens), which may limit the potential of VR-based training as they lack stereopsis, thus, limiting depth perception [11].

Current off-the-shelf head-mounted displays (HMD) that incorporate stereoscopic displays and head/body-tracking capabilities have shown their potential to provide a highly realistic visualization of the users’ real-time movements using avatars. Furthermore, like VR, Immersive VR (IVR) allows the creation of highly personalized virtual environments (VE) with an adaptable number of visual feedback elements [12]. In immersive VE, the use of avatars for self-representation is common and may have an important impact on user self-perception [13], interaction within the VE [14], and motor learning [15]. The use of avatars can allow for the visualization of one’s movements in real-time and has been implemented throughout various studies to teach movements by imitation learning [16–19]. Furthermore, the naturalistic and realistic visualization of VEs and avatars using HMDs may facilitate the transfer of the acquired skills in IVR to real-life applications [2].

Nonetheless, it is still an open question how immersive VEs should be designed to enhance motor learning. First, the perspective from which the user sees the VE might play a role in motor learning and performance. Virtual avatars can be visualized both in first-person perspective (1PP) or third-person perspective (3PP). In general, the 1PP has been reported to result in higher embodiment over avatars, i.e., in a higher sensation of being inside, having, and controlling a virtual body [20], than 3PP [21, 22] and thus it is generally the chosen perspective in different VR applications (e.g., [21–26]). Yet, the 3PP seems to provide better spatial awareness than the 1PP [22]. Nonetheless, when the relation between the person’s perspective and task performance has been analyzed, none of the perspectives have proved clear superiority over the other [21, 22, 27–29].

A second factor to evaluate when developing VEs for motor learning is the application of visual feedback elements. Motor learning literature suggests that augmented feedback – i.e., information obtained from “external” sources beyond our actions/movements [30] – is beneficial to enhance motor learning in the early stages of the training [1, 31]. Likewise, visual feedback can be provided during task execution (concurrent feedback) and/or after task completion (terminal feedback). Concurrent visual feedback has been shown to be especially effective in enhancing the learning of complex tasks [31, 32] – i.e., tasks that involve movements with several degrees of freedom and that require higher amounts of attention, memory, and processing capacity [1, 33] – and rather unfavorable for learning simple motor tasks, which benefit more from terminal feedback or a combination of both [31]. However, as a counterpart, concurrent visual feedback could also potentially increase the learner’s cognitive load [31], defined as the load that performing a certain task imposes on the learner’s cognitive system [34]. An excessive cognitive load may cause the learner to become overwhelmed, miss important details, and misunderstand information, thereby impeding learning [35].

In short, both the person’s perspective and the provision of visual feedback using HMD-IVR might affect motor learning, embodiment, and cognitive load. Another potential effect of the aforementioned factors is motion sickness. Cybersickness – or bodily discomfort caused by exposure to VR content – is a common downside effect of IVR use [36, 37] that could hamper the VR experience. On average, cybersickness affects 20% to 95% of users, depending on the immersive content [38]. Thus, it is important to know how the visual perspective and feedback provided in HMD-IVR systems affect cybersickness, as this will ultimately influence HMD-IVR systems’ usability.

In the field of motor rehabilitation, where the use of HMD-IVR has recently seen important growth (in various facets - e.g., upper-limb [39–44], lower-limb [40], and gait and balance rehabilitation [45–48] - and diverse populations - e.g., stroke [39–41, 46, 48], spinal cord injury (SCI) [42, 46], Parkinson’s disease [43, 46], multiple sclerosis [46, 48], and elderly people [47] -, understanding the implications of visual perspective and visual feedback to promote motor (re)learning becomes fundamental. Virtual rehabilitation interventions can be built to incorporate key features of motor learning, such as concurrent augmented feedback about movement patterns, as well as terminal feedback about movement performance to increase patients’ motivation [49].

Particularly in the emerging field of robotic gait rehabilitation, recent research has started evaluating the integration of feedback systems to enhance motor learning and performance [50, 51]. Especially, recent advancements in wearable lower-limb exoskeletons for gait rehabilitation and ambulatory assistance [52] have prompted research into integrating feedback systems to facilitate their use and boost recovery [51]. For users to successfully use this technology, they first need to undergo a long learning process on how to control the devices. The user needs to learn how to trigger steps, e.g., by shifting the weight from one leg to the other [53, 54], balancing [55, 56], and transitioning between sitting and standing positions [57]. This is especially challenging for individuals with sensorimotor disorders, such as SCI, who might also suffer from sensory loss, such as loss in proprioception, i.e., the position of the limbs in space. Therefore, the learning process to use a robotic exoskeleton becomes lengthy and tedious both for the user and the therapists accompanying the training [57–59], requiring not only physical but also high cognitive effort [60]. While efforts have been made to support the learning of using these devices, studies mainly focused on vibrotactile and electrostimulation feedback [61–66], not well suited for patients with sensory loss. The use of visual feedback might thus be better suited to support learning, also based on the literature that demonstrated that this is the most common method to support motor learning of complex tasks - e.g., in the fields of sports and neurorehabilitation [67–70].

Based on the aforementioned literature and identified clinical need, we created an HMD-IVR-based system to aid people in learning complex tasks, as is learning to operate a wearable exoskeleton for overground walking. When developing novel IVR environments for motor learning, it is necessary to first investigate the effect of augmented visual feedback and the perspective from which to visualize the virtual environment. Likewise, since people with sensorimotor disorders retain their cognitive abilities, it is preferable to evaluate the new technology with healthy participants rather than directly overloading patients and therapists performing exploratory experiments. Therefore, we ran a parallel-group feasibility study with forty healthy participants who trained to trigger vitual steps using an avatar either with or without concurrent augmented visual feedback and either from a 1PP or 3PP to answer the following questions:

1. Q1: Can users learn this complex task using a HMD-IVR-based system?
2. Q2: Does training with concurrent visual feedback enhance learning vs. training without visual feedback?
3. Q3: Does visualizing the VE from a 1PP enhance learning vs. visualizing the avatar from the 3PP?
4. Q4: Is there an interaction effect on learning between the provision of visual feedback and the person’s perspective?
5. Q5: How do the training factors (i.e., feedback and perspective, and their interaction) affect the participant’s experience; namely embodiment, cybersickness, usability, and workload?

We hypothesized that participants would enhance their performance after training with the HMD-IVR system (Q1). In particular, we expected a greater improvement in the ability to perform the task after training in participants receiving the concurrent visual feedback (Q2) and from 1PP (Q3), compared to training without feedback and from a 3PP, respectively. We did not have an a priori hypothesis regarding the interaction effects between the visualization perspective and concurrent feedback on motor learning (Q4), and therefore, this was treated as an exploratory evaluation. Finally, we expected 1PP to result in higher embodiment, usability, and cybersickness; and lower workload than 3PP (Q5). Likewise, receiving visual feedback was expected to increase usability and workload, while not affecting embodiment or cybersickness when compared to not receiving feedback. As before, the effect of the interaction on the participant’s experience was treated as an exploratory evaluation.

## Materials and methods

### Participants

Forty healthy participants (13 female, 27 male) without known motor or cognitive disorders and aged from 18 to 60 years old ($27.73 \pm 7.91$) participated in the study. Participants provided written informed consent to participate in the study and did not receive any compensation. The study was approved by the Human Research Ethics Committee of the Delft University of Technology (TU Delft) and conducted in compliance with the Declaration of Helsinki. The recruitment of participants was performed within the TU Delft via word-of-mouth and campus advertisement. Table 1 summarizes the participants’ demographics for each training modality, including gender, age, the highest level of education, and previous experience with VR and gaming.

**Table 1:** Participants demographics. Values are reported as median, minimum and maximum, and interquartile range (IQR)

Training modality	Training factors	Male	Female	Age (years)	Highest education level $^{a}$	Virtual reality experience $^{b}$	Videogames experience $^{c}$
NF	NO Feedback 1st PP	7	3	25 [19, 38] IQR = 3.50	4 [1, 4] IQR = 0	1 [0, 1] IQR = 1	5 [1, 7] IQR = 3.75
YF	YES Feedback 1PP	7	3	25 [18, 60] IQR = 6	4 [3, 4] IQR = 0.75	1.50 [0, 3] IQR = 1.75	4 [1, 7] IQR = 2.75
NT	NO Feedback 3PP	6	4	25 [21, 49] IQR = 4	4 [3, 4] IQR = 0	0.50 [0, 3] IQR = 1	4 [1, 7] IQR = 3
YT	YES Feedback 3PP	7	3	28.50 [23, 42] IQR = 7.25	4 [4, 4] IQR = 0	1 [0, 1] IQR = 1	3 [1, 7] IQR = 2.50

$^a$ Education level: 1 - “Less than primary”; 2 - “Primary”; 3 - “Secondary”; 4 - “Higher”

$^b$ Virtual reality experience: 1 - “0 h”; 2 - “1 h”; 3 - “15 h”; 4 - “more than 15 h”

$^c$ Videogames experience: from 1 (“Not at all”) to 7 (“Very much”)

### Virtual walking task

#### Experimental setup and virtual environment

**Fig. 1:**
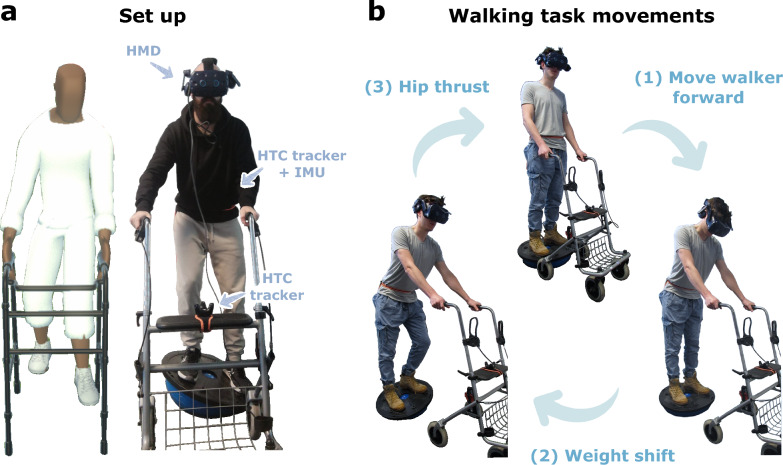
Experimental set-up and virtual walking task (**a**) The set-up consisted of an HMD, two HTC Vive trackers (placed on the participant’s pelvis and the walker), an IMU (placed on the participant’s pelvis), a balance board, and the walker. Participants’ movements were tracked (left) and imitated by the avatar in the virtual environment (right). (**b**) The virtual walking task consisted of triggering virtual steps by executing three consecutive movements that resembled those required to trigger steps in a wearable exoskeleton: (1) move the walker forward, (2) weight shift, and (3) hip thrust

The virtual walking task consisted of triggering virtual steps performed by a gender-neutral avatar (downloaded from the Unity Asset Store) visualized in the immersive VR using a commercial HMD (VIVE Pro 2 headset, HTC Vive, Taiwan & Valve, USA). In addition to the avatar, participants also visualized a virtual walker that mimicked the movements of a real 4-wheeled walker, which only allowed movements in the sagittal plane (Fig. 1a).

The avatar and walker were animated using the position and orientation of the HMD and two HTC Vive trackers, one attached to the participant’s pelvis at iliac crest level and the second one to the walker. To establish a connection between these components with the Unity software, we used the SteamVR plugin (version 2.7.3, Valve Corporation, USA). Likewise, the animation process was facilitated by using the Final IK package version 2.2 for Unity (Rootmotion, Estonia), which includes various inverse kinematics (IK) solvers and real-time procedural animation modification solutions. In addition, an inertial measurement unit (IMU) (Trigno Avanti Sensor, Delsys Inc., Boston, MA) was attached to the tracker on the pelvis to gather a more reliable measurement of the hip acceleration (i.e., hip thrust).

The avatar and virtual walker were scaled to match each participant’s (and walker’s) proportions. The walker scaling was performed by touching the top of the walker and pressing the HTC Vive controller’s button to record this position. The tracked height of the HMD was used to determine the scaling of the avatar. Before recording this position, we asked participants to stand up straight to make sure the height was recorded correctly.

Lastly, participants performed the virtual walking task while standing on a balance board (Bosu balance station, Domyos, Decathlon, France) to challenge their balance, enforcing them to rely on the walker. This resulted in an increased trunk inclination and ultimately causing fatigue in the arms, similar to what people with neurological disorders experience in real-life settings when learning to use a wearable exoskeleton.

The VE was developed using the Unity game engine (Unity Technologies, USA) version 2020.3.21, and ran with a framerate of 90 frames per second. The computer operated on Windows 10 Home 64-bit edition (Microsoft, USA) ran the task within the Unity Editor. The computer had 32 GB of DIMM DDR4 working memory, an NVIDIA GeForce RTX 3080 GPU, and an AMD Ryzen 5900X 3.70 GHz 12-Core processor (AMD, USA).

#### Step triggering

To trigger a (virtual) step, three consecutive movements needed to be successfully performed in sequential order (Fig. 1b):

***Movement 1: Move walker forward*** First, the participant needed to move the walker forward to create space such that the (virtual) leg did not collide with the walker. The distance the walker is moved forward determines the maximum possible stride length. If a step is successfully triggered (Movement 3) but a collision with the virtual walker would occur, the step will not take place.

***Movement 2: weight shifting*** Before the step could be triggered, the participant had to move the center of her/his pelvis laterally to match the center of the avatar’s leading leg, i.e., the foot currently positioned in front of the coronal plane, within a tolerance of 0.15 ms. This condition was required to trigger the step and had to be maintained until *Movement 3: Hip thrust* was achieved.

***Movement 3: Hip trust*** Once the participant moves the walker forward and accomplishes the weight shift, the participant can trigger the step by generating hip thrust, i.e., accelerating the hip in the anteroposterior direction. If the sequence of movements is performed correctly, participants can see the avatar moving the trailing leg (i.e., the leg whose foot is positioned behind the coronal plane) forward, performing a step. This stepping motion simulates the movement that would be generated by a wearable exoskeleton. Note that the real leg remains in place and, therefore, participants have to check the avatar’s leg position (if needed) to understand the current body configuration, as they cannot rely on their proprioception for this, thus emulating people with sensory loss who cannot rely on lower-limb proprioception.

To define these movements, we got inspiration from the movements that people with neurological disorders usually need to follow and learn to safely trigger steps when using a wearable exoskeleton for overground walking, e.g., weight shifting is commonly used as a control input to trigger steps [53, 54], and the hip thrust simulates the step intention, which can be used as a control input as well [71]. In fact, given that the robotic gait of people with neurological disorders requires essential postural adjustments and balance during the double support phase, each step can be considered as the commencement of gait. The biomechanical requirements for successful gait initiation are the generation of momentum (in the forward direction and in the direction of the trailing leg) and the maintenance of balance [72]. Therefore, the hip thrust movement provides a natural way to determine the user’s intention to initiate a step, while also actively involving the user in the decision to launch a step.

#### Stride length control

The triggered virtual stride length is determined by the peak pelvis acceleration $a_{peak}$ during hip thrust, measured with the IMU attached to the pelvis, according to the following linear relationship:

1 $$\begin{aligned} SL = {\left\{ \begin{array}{ll}\frac{a_{peak}}{a_{max}} \cdot SL_{max} & \text {if }{a_{max}} {>} {a_{peak}} {\ge } {a_{min}} \,{and} \,Hip_{Xdisp} \ge 2 \ cm \\ \\ SL_{max} & \text { if}\,{a_{peak}} {\ge } {a_{max}} \,\text { and}\,Hip_{Xdisp} \ge 2 \ cm \\ \\ 0 & \text {otherwise}, \end{array}\right. } \end{aligned}$$

where *SL* is the triggered stride length in meters (m). The peak acceleration ($a_{peak}$) is the highest acceleration reached by the participant during the hip thrust movement and measured by the IMU on the pelvis in the anteroposterior direction. The maximum acceleration ($a_{max}$) was fixed to $0.4\, \hbox {m} \cdot \hbox {s}^{-2}$ for all participants. In order to trigger a step, the hip’s peak acceleration needed to be higher than a predefined minimum acceleration ($a_{min}$ = $0.1\,$m$\cdot \hbox {s}^{-2}$), and the hip displacement in the anteroposterior axis ($Hip_{Xdisp}$) higher than 2 cm to prevent accidental triggers/steps. $SL_{max}$ is the participant’s predefined maximum possible stride length and is calculated by multiplying the participant’s optimal stride length ($SL_{opt}$) by a factor of 1.5. The value of this factor, as well as $a_{max}$, $a_{min}$, and $Hip_{Xdisp}$, were determined through an iterative process of experimentation by the researchers. This involved trial and error until identifying reasonable values that provided optimal comfort and were easily achievable through natural and comfortable movements.

The participant’s optimal stride length depends on their height and is calculated for each participant as:

2 $$\begin{aligned} SL_{opt} = \frac{1}{2} \cdot SL_{avg} \cdot BH, \ \ \ \end{aligned}$$

where *BH* is the participant’s body height and $SL_{avg} = 0.7774$ is the average stride length (in percentage of body height, %BH) obtained by Bovi et al. in healthy adults [73]. Therefore, we defined the optimal stride length as half the average of healthy adults, because people with sensorimotor loss tend to take shorter steps when walking with wearable exoskeletons [71, 74–77]. Furthermore, a shorter stride length might mitigate the occurrence of motion sickness by reducing the visually induced motion sickness (VIMS) - a subcategory of motion sickness that specifically relates to the perception of motion while remaining still [78].

In order to reduce step-by-step variation and maintain a constant stride length, we encouraged participants to keep the optimal stride length for every step. Note that the stride length needed to perform the optimal stride length – defined as *target* stride length ($SL_{target}$) – may vary depending on the previous stride length:

3 $$\begin{aligned} SL_{target} =\frac{1}{2} \cdot SL_{opt} + \left| Pos_{Leadingfoot}-Pos_{Trailingfoot} \right| . \end{aligned}$$

The target stride length ($SL_{target}$), thus, depends on the distance between the position of the trailing foot ($Pos_{Trailingfoot}$) and the leading foot ($Pos_{Leadingfoot}$) in the anteroposterior axis and the optimal stride length ($SL_{opt}$) calculated through Eq. 2.

### Training modalities

**Fig. 2:**
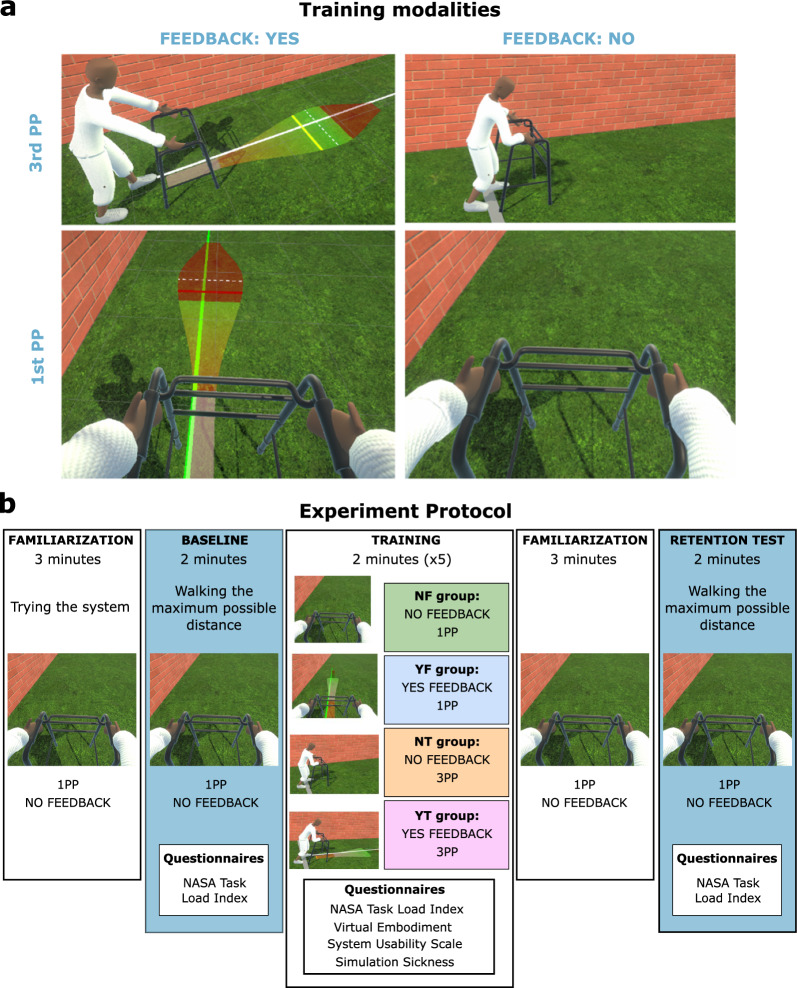
(**a**) The four training modalities. Each modality corresponds to a combination of two factors: concurrent visual feedback (ON or OFF) and visualization perspective (1PP or 3PP). (**b**) The experimental protocol followed a multi-arm pre-post design in which participants were randomly assigned to one of four training modalities

The experiment included four training modalities (Fig. 2a), each modality corresponding to combinations of two factors: visualization perspective (1PP or 3PP) and concurrent visual feedback (YES or NO).

#### Person perspective

Participants, based on the training modality, experienced the VE through two distinct perspectives: 1PP or 3PP (Fig. 2a). In the 1PP training modalities, the camera was positioned at the eye level of the avatar, offering participants a direct and immersive view aligned with the avatar’s visual field. In the 3PP modalities, the camera was situated laterally to the avatar’s position (approximately 4 m in lateral direction, raised by 1 m from the floor, and rotated 90 degrees to face the virtual avatar). This deliberate placement was chosen to optimize the visualization, ensuring participants had a comprehensive view of both the avatar and the visual feedback.

#### Visual feedback

**Fig. 3:**
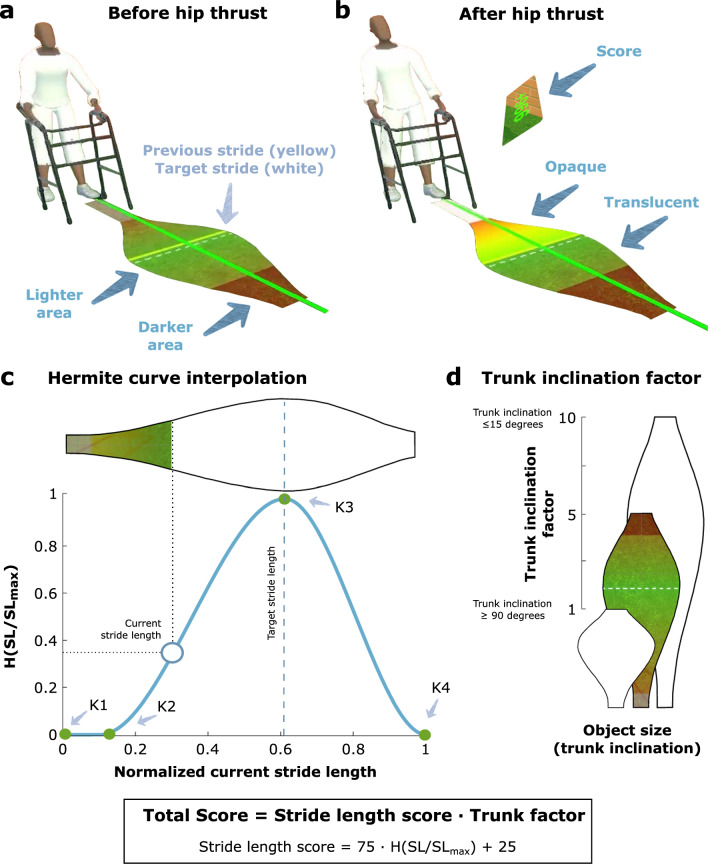
(**a**) Fusiform object before hip thrust movement. The border that separates the object into lighter and darker areas informs about the position of the walker relative to the trailing leg. (**b**) Fusiform object after hip thrust movement. The fusiform object, initially translucent, displays a dynamically changing opaque layer, which fills up to reflect the current peak acceleration until the maximum is reached. **(c)** Hermite curve interpolation with four keyframes *K*1, *K*2, *K*3, and *K*4 that defines the shape of the fusiform object and the stride length score ($SL \ score$ = 75 $\cdot H(SL/SL_{max})$ + 25; where $H(SL/SL_{max})$ corresponds to the value of the Hermite curve at the current stride length normalized over the maximum stride length). The stride length score ranges from 25 (minimum and maximum stride length) to 100 (target stride length). **(d)** Trunk inclination factor: the factor ranges from 1 (trunk inclination is $\ge$ 90 degrees) to 10 (trunk inclination is $\le$ 15 degrees). The total score, then, ranges from 25 to 1000

We aimed to design easy-to-understand and highly informative augmented visual feedback to support the learning of the different movements required to trigger a step. We attempted to achieve this by continuously *projecting* a fusiform object on the virtual floor in front of the avatar (Fig. 3a-b). The feedback provided by the virtual object is detailed in the following sections and summarized in Table 2. For a video of an experienced user demonstrating the virtual walking task and the visual feedback provided, see Additional file 1.

**Table 2:** Summary of the visual cues from the augmented visual feedback

Type	What?	Where?	How?
Movement 1: Move walker forward			
Concurrent	Maximum stride length possible due to relative walker position	Border that separates the object into a lighter and a darker area	Indicates the maximum stride length that participants can reach without colliding with the walker. If the stride length is longer than the distance between the trailing leg and the walker, it will produce a walker collision, and the step will not occur.
Concurrent	Trunk inclination	Size of the object	When there is no inclination, the length of the bar is maximum, and vice versa.
Movement 2: Weight shifting			
Concurrent	Weight shifting	Lateral position of the object	If the participant moves the pelvis to the right (left) (and, thus, the avatar too), the object moves to the right (left). When the lateral positions of the centerline of the fusiform object and the leading foot match, the longitudinal white line displayed in front of the leading foot turns green and indicates that the weight shifting is accomplished. The step trigger is allowed now.
Movement 3: Hip thrust			
Concurrent	Target stride length	White dashed line	The target stride length depends on the optimal stride length and varies depending on the current absolute distance between the feet in the anteroposterior direction.
Concurrent	Target stride length	Color and width of the opaque object	The widest part of the object and the green color are centered on the target stride length. The thinnest part of the object and the red color are on the extreme points of the object.
Terminal	Previous stride length	Yellow line	Helps the participants to know the acceleration they did to perform the previous stride length.
Concurrent	Stride length	Object turning opaque	The higher the peak hip acceleration, the more part of the object turns opaque and the larger will be the stride length.
Concurrent	Minimum stride length	Bottom narrow white rectangle	The step trigger is not activated unless participants generate enough hip acceleration to pass the rectangle, which becomes white during the hip thrust.
Other			
Terminal	Score	Pop-up window	The score is based on the trunk inclination and the deviation from the target stride length.

### Concurrent and terminal visual feedback

#### Concurrent feedback related to maximum stride length possible due to relative walker position

The position of the walker relative to the trailing leg is indicated in the fusiform object as the border that separates the object into lighter and darker areas, where the darker area is located towards the end of the object (Fig. 3a). Note that due to the scaling factor applied to the object in the longitudinal direction, the position of this border is proportional to the distance between the walker and the trailing leg, but does not necessarily match the actual walker position. The position of this border w.r.t. the participant indicates the maximum stride length that participants can reach without colliding with the walker. We determined the position of this border by normalizing the distance between the walker and the trailing leg with the maximum stride length $SL_{max}$, i.e., the closer the walker to the trailing leg, the smaller the possible stride length, and the closer the border to the participant. Therefore, if the stride length of the triggered step was longer than the distance between the trailing leg and the walker, it would result in a collision with the virtual walker. Thus, when this was the case, the step was not triggered on the avatar.

#### Concurrent feedback related to trunk inclination

The position of the walker might also affect the trunk inclination, i.e., the further the walker is in front of the participant, the larger might be the trunk inclination. To inform participants on their trunk inclination as a means to reduce it, we employed the length of the fusiform object in the anterior direction (Fig. 3d) – i.e., when the trunk inclination is $\le$15 degrees, the length of the object is maximum (length = 2.0 m), and when the trunk inclination is $\ge$90 degrees, the length of the object is minimum (length = 0.3 m). Note that trunk inclinations below 15 degrees did not affect the length of the fusiform object to avoid excessive size changes when standing up. Nevertheless, values below this threshold were still recorded for later analysis (see Section Data processing).

#### Concurrent feedback related to weight shifting

A longitudinal white line is displayed on the floor in front of the leading foot, i.e., the foot positioned in front of the coronal plane (left leg in Fig. 2a). The lateral position of the centerline of the fusiform object w.r.t. the participant’s sagittal plane shows the lateral position of the pelvis, i.e., if the participant moves the pelvis to the right (left) w.r.t the sagittal plane, the object moves to the right (left). When the lateral positions of the centerline of the fusiform object and the leading foot match, the longitudinal line displayed in front of the leading foot turns green (Fig. 3a, b). This means that the weight shift (Movement 2) is accomplished, and the step can be triggered with the hip thrust (Movement 3).

#### Concurrent feedback related to optimal stride length

The visual information regarding the target stride length was provided to participants by modulating the shape of the fusiform object using a piecewise cubic Hermite interpolation (achieved in Unity using the *AnimationCurve* class) to interpolate between key points smoothly. An example of the shape of this curve can be seen in Fig. 3c. We defined this curve using four keyframes, namely a start keyframe (*K*1 = (0, 0)), a keyframe to indicate the minimum stride length ($K2=\left( SL_{min}/SL_{max}, 0\right)$), a keyframe to indicate the target stride length ($K3 = \left( SL_{target}/SL_{max}, 1\right)$), and an end keyframe ($K4 = \left( 1, 0\right)$) representing the maximum stride length ($SL_{max}$). Furthermore, we set the tangents (derivatives) of the four keyframes to zero.

The *x*-position of *K*3 in the curve indicates the target stride length ($SL_{target}$), and we calculate it by normalizing the target stride length over the maximum possible stride length ($SL_{max}$) that corresponds to the maximum acceleration ($a_{max}$). The *x*-position of *K*2 indicates the minimum stride length ($SL_{min}$) and corresponds to the minimum acceleration ($a_{min}$) required to trigger a step (see also Fig. 3b). Once again, we calculate the *x*-position of this keyframe by normalizing this value w.r.t. the maximum stride length. Finally, the $H(SL/SL_{max})$ in Fig. 3c is the value of the Hermite function that depends on the *x*-position, i.e., the current stride length normalized w.r.t. the maximum stride length.

As a result, the curve is the smallest at the base (spanning from *K*1 to *K*2) and at the end keyframe (Fig. 3c). Likewise, the position of the widest part of the object (*K*3) can vary in each step as we calculate it using the actual relative distance between both feet (see Eq. 3). Furthermore, the fusiform object is filled by a color gradient, with green on the wider part and red at the object’s extremes. The narrow base of the object (ending at *K*2,) is colored white to indicate the area in which no step will be triggered because $a_{min}$ was not reached.

The fusiform object, initially translucent, displays a dynamically changing opaque layer, which fills up to reflect the current peak acceleration until the maximum is reached (Fig. 3b). When this opaque layer surpasses the white base, which corresponds to the minimum stride length, a step is triggered. The object also features a dashed white line at its widest area, indicating the target stride length (Fig. 3a). Furthermore, the object contains a yellow line, representing the previous stride length normalized over $SL_{max}$ (terminal feedback; Fig. 3a). This visual aid encourages participants to maintain optimal stride length in subsequent steps based on their experience from the previous one.

The fusiform object includes a darker area near its end, whose starting point represents the position of the walker w.r.t. the trailing leg (see subsection *Feedback related to maximum stride length possible due to relative walker position*). If a step is to be landed within this darker area, a collision with the walker would occur. Therefore, a step must land between the threshold at the base and the border of the darker area to successfully be triggered.

#### Terminal feedback: Score

Participants who trained with visual feedback also received terminal feedback on their performance after each step to motivate and encourage them to enhance their performance. A pop-up window appeared in front of the avatar after each step with a score obtained for that step (Fig. 3b). The score is based on the trunk inclination and the deviation from the target stride length of each step following the equation:

4 $$\begin{aligned} Score = SL \ score \cdot Trunk \ inclination \ factor, \end{aligned}$$

where $SL \ score$ is the score related to the stride length (see Eq. 5) and the $Trunk \ inclination \ factor$ is a value that ranges linearly from 1 –when the trunk inclination is $\ge$ 90 degrees– to 10 –when the trunk inclination is $\le$ 15 degrees (Fig. 3d). Note that the trunk inclination is a continuous variable. The $SL \ score$ depends on the value of the Hermite curve corresponding to the current stride length normalized over the maximum stride length $SL_{max}$ (see subsection *Movement 3: Hip trust* and Fig. 3c) following the equation:

5 $$\begin{aligned} SL \ score = 75 \cdot H(SL/SL_{max}) + 25. \end{aligned}$$

Thus, the stride length score ranges from 25 (corresponding to the minimum and maximum stride lengths, i.e., $SL_{min}$ and $SL_{max}$) to 100 points (target stride length, i.e., $SL_{target}$). The total score, then, ranges from 25 to 1000. A minimum score of 25 was decided to prevent participants from receiving zero points that might hamper their motivation, ensuring that they would always receive at least this amount in the worst-case scenario. Note that the score was only shown once the step was triggered.

### Experiment protocol

The experiment protocol followed a multi-arm pre-post study design (Fig. 2b) where we assigned participants randomly to one of four training modalities, with ten participants per condition, each modality corresponding to combinations of two factors: concurrent visual feedback (YES or NO) and visualization perspective (1PP or 3PP). The experiment was conducted collaboratively by a technical developer of the project and a support person not involved in the developmental phase.

Before starting the experiment, participants received theoretical training on the virtual walking task. We gave participants time to read the instructional slides (see Additional file 2) on a computer screen and ask questions if needed until they felt prepared. All participants were informed that their performance would be evaluated based on three sub-tasks: 1) their ability to walk the maximum distance possible (i.e., ability to trigger steps) while 2) maintaining an upright posture and 3) an efficient stride length (i.e., not too short, not too long). Further questions were allowed during the experiment except when performing the baseline and retention tests. Importantly, the research team in charge of the experiment only provided (or reminded) information that was in the instructional slides. After being briefed on the experiment objectives, instructions, and task details, participants answered an initial set of demographic questions (Table 1).

After the theoretical training, participants conducted a 3-minute familiarization phase, in 1PP and without feedback, to allow them to try the system and accustom themselves to the VE. After the familiarization, the experiment began with a baseline test. During baseline (and retention tests), we asked participants to virtually “walk” with the avatar the maximum distance possible, following the aforementioned instructions. During baseline, familiarization, and retention tests, participants observed the VE in 1PP and without concurrent visual feedback since this is the closest to the natural way we walk and experience the real world.

After the baseline test, the training phase started. This phase consisted of five trials of two minutes each, where participants trained to improve their performance under the training modality to which they were assigned. Before starting the training, participants allocated to the conditions with concurrent visual feedback received additional theoretical training on the different elements of the visual feedback (see Additional file 3). This training was presented in the same way as the instructional slides at the start of the experiment. Note that the score was shown only during the training and only for modalities with feedback. Participants were allowed to take brief breaks ($\le$ 5 min) between trials to ask questions or take a rest.

After the training, we asked participants to answer four questionnaires to evaluate the embodiment they felt over the avatar, the usability of the system, the cybersickness experienced (if any), and the perceived workload (see Section *Data analysis*). The workload was also assessed after both the baseline and the retention tests. The questionnaires were filled out electronically in English and inside Unity using the VR Questionnaire Toolkit [79].

After answering the questionnaires, all participants carried out a second familiarization period of three minutes. This (re)familiarization aimed to wash out participants’ recent experience with the task environment and reduce any immediate aftereffects of training conditions on the performance. The retention test, which had the same form as the baseline test, was performed right after this (re)familiarization.

### Outcome measures

We recorded the participants’ head and hip positions and orientations using the HMD and the HTC Vive trackers located on the hip and walker. The acceleration of the hip was recorded at all times by the IMU. The data processing was performed in MATLAB (MATLAB R2021b, The MathWorks Inc., Natick, MA, USA).

#### Motor learning

In evaluating the learning process, we discerned two key aspects: the sequence involving the initiation of steps, reflected in the number of steps performed (main outcome), and the quality of the sub-tasks sequence (secondary outcomes), reflected in trunk inclination and stride length. These aspects required participants to learn and train on the three distinct sub-tasks: triggering a step, controlling trunk inclination, and controlling stride length.

**Main outcome** The *number of steps* - the result from triggering steps effectively - was chosen as the main metric to assess learning, with a higher number of steps indicating greater proficiency and learning.

**Secondary outcomes** We used the trunk inclination and deviation from the target stride length metrics to assess the quality/technique of the steps that were triggered. The *trunk inclination* was estimated by the angular deviation of the segment that connects the HMD with the tracker on the hip and the calibrated vertical when the participant stood completely upright. We averaged the *trunk inclination* during the entire test. Note that good performance is associated with small trunk inclinations because an increased trunk inclination indicates that the participant is relying excessively on the walker.

Stride length, defined as the distance between the point of initial contact of one foot with the floor and the point of initial contact with the floor of the same foot, was recorded for each step directly from Unity. The *deviation from the target stride length* was then calculated as the average percentage difference between the participant’s stride length and the participant’s target stride length in absolute value. This outcome metric was calculated from all the steps performed during the test and averaged through the test.

#### Questionnaires

The impact of the visual feedback and perspective on participants’ experience was assessed using the following outcome metrics:

**Embodiment** To assess the level of *embodiment* over the avatar, we selected several statements from the well-established embodiment questionnaire in [80, 81] and adapted them for our application. The questionnaire consisted of six statements to assess all three embodiment components, namely, body ownership – i.e., one’s self-attribution of a body –, (self-)location – i.e., volume in space where one feels to be located –, and agency – i.e., feeling in control of own movements [20, 80]. Since the number of questions related to each component was different, we weighted them to ensure equality. Participants responded on a Likert scale between 1 and 7 points; 1 indicated “Strongly disagree” and 7 indicated “Strongly agree”. The statements, their weight during analysis, and their targeted component of embodiment can be found in Additional file 4.

**Usability** The System Usability Scale [82] (SUS) was employed to evaluate the *usability* of the four different training modalities. The SUS has been widely used to assess the usability of software and hardware solutions [83, 84] and measures different aspects such as efficiency, effectiveness, and satisfaction. The questionnaire consists of 10 questions (see Additional file 4) with five response options on a Likert scale; 1 indicated “Strongly disagree”, and 5 indicated “Strongly agree”.

**Cybersickness** Although the Simulator Sickness Questionnaire (SSQ) was initially intended for simulator sickness assessment [85], it is also currently employed for *cybersickness* assessment [86]. The questionnaire prompts participants to provide subjective severity ratings of 16 symptoms on a four-point scale (none = 0, slight = 1, moderate = 2, severe = 3) after the exposure to the system [85]. These symptoms can be classified into three categories: Oculomotor, disorientation, and nausea [85]. Each category has its own score and is defined as the sum of its symptom scores multiplied by a constant scaling factor. In addition, there is a total simulator sickness score (TS) to obtain a single score, which is calculated by adding the raw scores (i.e., without the individual scaling factor) of the three categories and multiplying by a constant factor [85, 86]. Additional file 4 contains information on the symptoms and how to compute the scores.

**Workload** To measure the overall *workload* while using the IVR system, we employed the widely accepted and validated Raw Task Load Index (RTLX) – the most common adaptation from the NASA Task Load Index [87] in which the weighting process is omitted [88]. The workload is calculated by asking participants to graphically indicate their perceived cognitive demand (low/high or good/poor) on a response scale of 21 marks across six dimensions, namely mental, physical, and temporal demands; performance; effort; and frustration. The total score is computed by adding the score of each question and dividing it by six. The questionnaire can be found in Additional file 4.

### Statistical analysis

Normality was assessed using Shapiro-Wilk’s normality test, and homogeneity of variances was assessed by Levene’s test. To detect outliers, boxplots were examined, and extreme outliers – values exceeding $Q3 + 3\cdot IQR$ or falling below $Q1 - 3\cdot IQR$ – were identified and removed from all metrics. In these expressions, *Q*1 is the first quartile (25th percentile), *Q*3 is the third quartile (75th percentile), and the *IQR* refers to the interquartile range, which is the difference between *Q*1 and *Q*3. Additionally, two participants were excluded from the analysis of the deviation from the target stride length, as neither succeeded in taking a single step during the baseline test. Statistical analyses were carried out using *R* version 4.2.0, and the significance level was set to $\alpha$ = 0.05.

We used one-way analysis of variance (ANOVA) to verify that potential confounding variables such as age, level of education, experience using VR, and experience using video games were fairly balanced (by randomization) across the groups. When the one-way ANOVA assumptions were violated, the Kruskall Wallis rank sum test was applied.

To evaluate whether, overall, participants significantly improved their gait performance - i.e., number of steps (main outcome), and trunk inclination and deviation from target stride length (secondary outcomes) - from baseline to retention, paired *t*-tests in the case of normally distributed data or paired Wilcoxon signed-rank tests for non-normal distributed data were employed for each condition.

To evaluate whether participants improved their gait performance differently depending on the training condition they were allocated to, we employed a two-way ANOVA with the main and secondary outcomes change from baseline to retention (i.e., the difference between the retention values and the baseline values) as dependent variables and with independent values the type of visual *feedback* (ON vs. OFF), the *perspective* (1PP vs. 3PP), and their interaction [89]. When the two-way ANOVA assumptions were violated, the robust two-way ANOVA (using the *WRS2* package from *R*) was employed [90]. In the case of statistically significant interactions in the two-way ANOVA, posthoc pairwise comparisons with Tukey corrections were performed to compare levels of factors.

Regarding the questionnaires, a single value per questionnaire (and per subcomponents of the questionnaire) and per participant was computed following their specific conventions and utilized for the analysis. A two-way ANOVA was used to examine the main effect of the visual feedback condition and the perspective, and their interaction on the embodiment, usability (SUS), and cybersickness (SSQ) questionnaire answers collected after the training period. In the case of statistically significant interactions, posthoc pairwise comparisons with Tukey corrections were performed. Again, robust two-way ANOVA was used if the ANOVA assumptions were violated.

The participants’ cognitive load was subjectively measured using the RTLX questionnaire at three different time points, namely after baseline (B), after training (T), and after the retention test (R). A linear mixed-effects model (LMM) with participants as a random effect (see Eq. 6) was used to investigate the effect of *time*.

6 $$\begin{aligned} dv \sim feedback \cdot perspective \cdot time; random = \ \sim 1|ID, \end{aligned}$$

where *dv* is the dependent variable, *feedback*, *perspective*, and *time* are the fixed-effects, and *ID* is the participant identification and the random-effect. The LMM has no random slopes as indicated by $\sim 1$.

## Results

### Demographic factors

We found no significant differences between groups in terms of age, level of education, experience using VR, and experience using video games (Table 1). Therefore, no confounding effects are expected.

### Q1: Overall motor learning

In general, all participants significantly increased the number of steps after the training period, independently of the perspective and the visual feedback provided (Table 3). However, participants did not change their average trunk inclination or the deviation from the target stride length from baseline to retention, independently of the perspective and the visual feedback provided.

**Table 3:** Results from *t*-test statistical comparison of gait metrics between baseline and retention tests. Bold highlighting indicates statistical significance (***p<0.001, **p<0.01, *p<0.05)

Factor			Number of steps			Trunk inclination (deg)			Deviation from the target stride length (%)		
			Value	Effect size	Sig.	Value	Effect size	Sig.	Value	Effect size	Sig.
Overall		Baseline	6.95±5.32	0.80	W = 11, n = 40, ** p** $<$0.001 ***	20.13±6.70	0.00	t(39) = 0.00, p = 0.998	28.49±8.67	0.18	t(37) = 1.12, sparabreak p = 0.270
		Retention	13.32±5.54			20.12±8.79			26.33±7.40		

### Q2 & Q3: Main effects of visual feedback and person perspective on motor learning

**Fig. 4:**
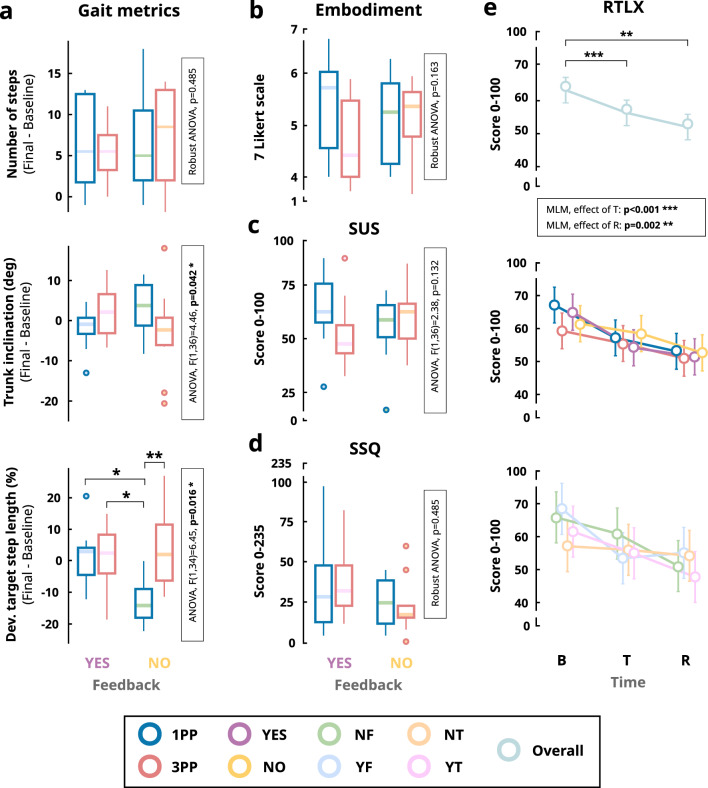
(**a**) Boxplot of the difference between the performance in baseline and retention tests of the gait metrics (number of steps, trunk inclination, and deviation from the target stride length). (**b**) Boxplot for the Virtual Embodiment questionnaire. (**c**) Boxplot for the SUS questionnaire. (**d**) Boxplot for the SSQ questionnaire. (**e**) Interaction plot involving mean values and standard deviation for the RTLX questionnaire by overall, factors (perspective: 1PP and 3PP; feedback: YES and NO), and training modality with respect to the time (B: baseline test, T: training, R: retention test). White rectangles show the statistical results of the interaction. Asterisks show the posthoc analysis results: ***p<0.001, **p<0.01, *p<0.05

We found a significant main effect for the perspective on the deviation from the target stride length from baseline to retention (Table 4 and Fig. 4a), where 1PP seemed to reduce it compared to 3PP. However, we found no significant main effects of either perspective or visual feedback on the increase in the number of steps or the difference in trunk inclination.

**Table 4:** Results from the (Robust) two-way ANOVA statistical analysis on the change of performance gait metrics and Embodiment, Usability (SUS), and Cybersickness (SSQ) questionnaires. Bold highlighting indicates statistical significance (***p<0.001, **p<0.01, *p<0.05)

Metric		Significance		
		Feedback	Perspective	Interaction
Gait metrics	Number of steps $^a$	*p* = 0.764	*p* = 0.867	*p* = 0.485
	Trunk inclination $^b$	*F*(1,36) = 0.00, ${\eta _{g}}^{2}$ = 0.00, *p* = 0.996	*F*(1,36) = 0.17, ${\eta _{g}}^{2}$ = 0.01, *p* = 0.686	***F*(1,36) = 4.46,** ${\eta _{g}}^{2}$ **= 0.11,** p = 0.042 *
	Deviaton from the target stride length $^b$	F(1,34) = 2.39, ${\eta _{g}}^{2}$ = 0.07, *p* = 0.132	***F*(1,34) = 5.96,** ${\eta _{g}}^{2}$ **= 0.15,** p = 0.020 *	***F*(1,34) = 6.45,** ${\eta _{g}}^{2}$ **= 0.16,** p = 0.016 *
Virtual embodiment	Total score $^a$	*p* = 0.742	*p* = 0.377	*p* = 0.163
	Body ownership $^b$	*F*(1,35) = 0.01, ${\eta _{g}}^{2}$ = 0.00, *p* = 0.946	*F*(1,35) = 3.61, ${\eta _{g}}^{2}$ = 0.09, *p* = 0.066	*F*(1,35) = 0.64, ${\eta _{g}}^{2}$ = 0.02, *p* = 0.428
	Location $^b$	*F*(1,35) = 0.19, ${\eta _{g}}^{2}$ = 0.01, *p* = 0.663	***F*(1,35) = 9.44,** ${\eta _{g}}^{2}$ **= 0.21,** p = 0.004 **	*F*(1,35) = 0.81, ${\eta _{g}}^{2}$ = 0.02, *p* = 0.373
	Agency $^a$	*p* = 0.644	*p* = 0.185	*p* = 0.087
SUS $^b$		*F*(1,36) = 0.01, ${\eta _{g}}^{2}$ = 0.00, *p* = 0.924	*F*(1,36) = 0.393, ${\eta _{g}}^{2}$ = 0.01, *p* = 0.535	*F*(1,36) = 2.38, ${\eta _{g}}^{2}$ = 0.06, *p* = 0.132
SSQ	Total score $^a$	*p* = 0.148	*p* = 0.930	*p* = 0.485
	Nausea $^a$	*p* = 0.526	*p* = 0.899	*p* = 0.377
	Oculomotor $^a$	*p* = 0.452	*p* = 0.933	*p* = 0.361
	Disorientation $^a$	p = 0.023 *	*p* = 0.486	*p* = 0.375

$^a$ Robust two-way ANOVA on gain scores (trimmed means). Trimming level = 20%

$^b$ Two-way ANOVA on gain scores

### Q4: Interaction effects of visual feedback and person perspective on motor learning

We found a significant interaction between perspective and visual feedback in trunk inclination from baseline to retention (Table 4 and Fig. 4a). In particular, when the feedback was provided during training, participants slightly reduced their trunk inclination when training with 1PP, but slightly increased it with 3PP. However, when they trained without feedback, the 3PP group showed a slight reduction in the trunk inclination, while the 1PP exhibited a slight increase. We also found a significant interaction for the deviation from the target stride length. In particular, training without feedback and 1PP (i.e., NF group) significantly outperformed the other groups (Additional file 5).

### Q5: Effects of training factors on user experience

#### Embodiment

Participants reported, on average, a high sense of embodiment (i.e., total score) during the training period independently of the perspective and feedback received (NF = $5.12 \pm 0.87,$ YF = $5.39 \pm 0.98,$ NT = $5.12 \pm 0.74,$ YT = $4.71 \pm 0.84$; score range = [1, 7]; Fig. 4b). When analyzing the embodiment subscales, we found a main effect of the perspective in the *Location* component (Table 4). In particular, the embodiment scores were higher for participants training in the 1PP compared to those who trained with 3PP (1PP = $5.75 \pm 1.33,$ 3PP = $4.50 \pm 1.57$).

#### Usability

We found that the HMD-IVR-based training system was rated marginally acceptable (SUS; NF = $55.00 \pm 17.04,$ YF = $63.50 \pm 17.45,$ NT = $59.75 \pm 14.88,$ YT = $52.25 \pm 16.09$; score range = [0, 100]; Fig. 4c), according to the terms assessed by Bangor et al. to evaluate the acceptability of a system when using the SUS [91]. We did not find significant main effects of the perspective, the visual feedback, or their interaction on the usability (Table 4).

#### Cybersickness

The total score from the SSQ showed relatively low scores in all of the training modes (NF = $24.31 \pm 15.89,$ YF = $33.66 \pm 29.40,$ NT = $22.07 \pm 17.67,$ YT = $36.65 \pm 20.99$; score range = [0, 235.65]; Fig. 4d). We did not find significant main effects of the perspective nor the visual feedback and their interaction in most of the subscales, except for a main effect of the visual feedback on the Disorientation scores (Table 4). In particular, participants who trained with the visual feedback reported significantly higher values in the Disorientation subscale than those without feedback (YES = $38.28 \pm 30.25,$ NO = $18.10 \pm 24.78$; score range = [0, 292.32]).

#### Perceived workload

We found that the time at which the questionnaire was completed (B: baseline, T: training, R: retention) had a significant effect on the RTLX score (Table 5 and Fig. 4e). In general, participants reported significantly lower cognitive load after the training than at baseline (B = $63.20 \pm 12.45,$ T = $56.30 \pm 12.55$; score range = [0, 100]), and at the final test (F = $52.05 \pm 12.05$) than at baseline.

**Table 5:** Results from the linear mixed-effects model (LMM) statistical analysis of the RTLX questionnaire. Only significant results ($p<0.05$) and tendencies ($p<0.1$) are shown in this table. The complete analysis can be found in Additional file 6. Bold highlighting indicates statistical significance (***p<0.001, **p<0.01, *p<0.05)

RTLX	Effect	Significance	Model effect size
Total Score	Time (Training)	***b*** = −3.00 (95% CI: −4.60, −1.40), *t*(72) = −3.54, *p* < 0.001 ***	1.10
	Time (Retention)	***b*** = −2.67 (95% CI: −4.27, −1.06), *t*(72) = −3.14, *p* = 0.002 **	
Mental demand	−	−	0.83
Physical demand	Time (Training)	***b*** = −3.20 (95% CI: −5.13, −1.27), *t*(72) = −3.13, *p* = 0.003 **	1.16
	Feedback * Training	b = 3.90 (95% CI: 1.17, 6.63), t(72) = 2.70, p = 0.009 **	
Temporal demand	−	−	0.86
Performance	Time (Training)	***b*** = −6.70 (95% CI: −9.46, −3.94), *t*(72) = −4.59, *p * < 0.001 ***	1.00
	Time (Retention)	***b*** = −4.90 (95% CI: −7.66, −2.14), *t*(72) = −3.36, *p* = 0.001 **	
	Perspective * Training	***b*** = 4.90 (95% CI: 1.00, 8.80), *t*(72) = 2.38, *p* = 0.020 *	
Effort	Perspective	***b*** = −2.80 (95% CI: −5.31, −0.29), *t*(36) = −2.14, *p* = 0.038 *	1.06
	Time (Training)	***b*** = −3.40 (95% CI: −5.23, −1.57), *t*(72) = −3.51, *p* < 0.001 ***	
	Time (Retention)	***b*** = −3.20 (95% CI: −5.03, 1.37), *t*(72) = −3.30, *p* = 0.002 **	
	Perspective * Training	***b*** = 2.80 (95% CI: 0.21, 5.39), *t*(72) = 2.04, *p* = 0.045 *	
Frustration	−	−	0.81

The analysis of the RTLX subcomponents showed a significant main effect of time on the *Effort* and *Performance* components, where participants reported less effort (mental and physical) and higher perceived performance in the final test than at baseline. Furthermore, these subcomponents showed significant interactions after the training test between perspectives. In particular, participants walking in 1PP experienced a greater reduction in effort and a higher perceived performance from baseline to the training test. Similarly, participants training with the visual feedback showed a greater reduction in the Physical demand subcomponent from baseline to training test than participants training without feedback.

## Discussion

The purpose of this study was to investigate how the person’s perspective (1PP vs. 3PP) and the addition of visual feedback in IVR influence the learning of complex motor tasks such as learning to control a virtual lower-limb exoskeleton. Additionally, we assessed the effect of these two factors on the user’s experience, namely embodiment, usability, cybersickness, and perceived workload. In discussing our findings, we return to the five research questions stated in the “Introduction” that we aimed to answer with our experiment.

### Participants can learn to trigger steps, but training has no effect on reducing trunk inclination nor on learning the “optimal” stride length

In general, all participants significantly increased the number of steps after training. This outcome partially supports our initial hypothesis. Indeed, on average, participants were able to improve the number of steps significantly in as little as ten minutes of practice (Additional file 7). However, this improvement in the number of steps is not accompanied by an improvement in the secondary performance metrics, namely trunk inclination and the deviation from the target stride length.

The complexity of the task, which required learning the coordination of several body movements, together with the potentially intricate nature of the visual feedback, probably played a role in the observed limited improvements on the secondary outcomes. This is supported by the observed high participants’ perceived workload, which despite being reduced over time remained high through the experiment, and the marginally acceptable usability scores. Since the allocated time for training was rather short, we speculate that participants mainly remained in the first cognitive stage of motor learning, where the optimization of physical effort, i.e., reduction of trunk inclination and constant stride length to reduce effort and fatigue, is not a priority. The minimization of effort is usually observed later on in the learning process, in the so-called autonomous stage [92].

### Training with concurrent visual feedback does not seem to enhance the learning of this particular complex task

Contrary to our hypothesis, the visual feedback does not seem to support the learning of the complex task. This is in contrast to the abundant literature that supports the benefit of concurrent visual feedback on motor learning [1, 31], especially when training complex tasks. A possible explanation for the limited effectiveness of the designed visual feedback is that, despite our efforts, the fusiform object was too complex to be interpreted by the participants in the short allocated time for training. It has been shown that too much superimposed visual information may overload the learner with excessive information, resulting in participants not being able to focus on the task [93]. This aligns with Wickens (2002) multiple-resource theory, which states that distributing information across different feedback modalities -e.g., visual, haptic, or auditory- is more effective than presenting the same amount of information within a single modality [94]. This is supported by the work of Oviatt et al., which showed that users prefer multimodal to unimodal feedback modality in especially complex tasks that require increasing cognitive demands [95]. Yet, we did not find significant differences in the perceived workload between participants trained with visual feedback w.r.t. those who trained without visual feedback. Future work should focus on designing other forms of feedback, e.g., haptic or multimodal feedback, which have been shown to be beneficial in training especially complex tasks [1], or to train individual sub-tasks consecutively to reduce the amount of conveyed information [96].

### The person’s perspective does not seem to affect the learning of this complex task

Contrary to our hypothesis, we found that the visualization perspective had no effect on the improvement of the number of steps and the trunk inclination. Nonetheless, we found a significant main effect of the perspective on the deviation from the target stride length. This difference seems to be mostly explained by the significant interaction effect between perspective and feedback, i.e., the first-person perspective and no visual feedback group improved the deviation from the target stride length to a significantly greater extent than participants from the other groups - a topic that will be further discussed in the subsequent subsection.

Thus, our results are in line with previous literature that did not find a clear superiority of one perspective over another when the relation between the person’s perspective and task performance was analyzed [21, 22, 27–29]. Yet, other studies demonstrated that the first-person perspective enables more accurate interactions in the VE [22]. Likewise, based on the work of Gorisse et al. [22], one could have expected that the third-person perspective would support reducing the trunk inclination, as it has been shown to provide better spatial awareness. One possible explanation for the lack of superiority of the third- over the first-person perspective is that participants in the third-person perspective might have focused more on the movements of the avatar’s legs and less on the trunk inclination in an effort to reduce the complexity of the task by prioritizing triggering steps (the main goal of the task) over improving posture. However, we are unable to verify this hypothesis with the data collected. Future research should aim to investigate this aspect further, potentially by incorporating eye-tracking data to assess where participants’ attention is directed.

### There is no single combination of visualization perspective and visual feedback that enhances the learning of all sub-tasks

While we did not find a significant reduction in trunk inclination between baseline and retention tests for participants overall, we found interaction effects between the person’s perspective and the visual feedback on changes in the trunk inclination from baseline to retention.

As discussed above, the potentially increased spatial awareness of the user’s posture provided by the third-person perspective [21, 22, 97] might have facilitated the reduction of the trunk inclination after training when no visual feedback is provided. Thus, the addition of visual feedback may be redundant, superimposing even more visual information to the already informative visual feedback. The contrary might be happening when training in a first-person perspective. In that case, as participants do not directly see the inclination of the avatar’s trunk, they need to rely on visual information to improve this metric.

Regarding the deviation from the target stride length metric, we also found interaction effects between the person’s perspective and the visual feedback. In particular, only participants who trained with the first-person perspective and without visual feedback showed significant improvements in this metric. This result was unexpected, as the only information on the stride length that these participants received was the location of their legs. Here, it is important to note that the baseline and retention tests were performed in first-person perspective, as this better aligns with performing the task in the real world. Therefore, based on the specificity-of-learning hypothesis [98], those participants who trained with a first-person perspective and without visual concurrent feedback, might have had an advantage over those who trained with a third-person perspective, as their training condition matched that of the retention test. While we added a (re)familiarization phase between training and retention to washout this effect, it cannot be ruled out that some residual effects of perspective and feedback differences may have influenced retention scores. A second explanation for this observation could be that participants in this group might have focused primarily on improving the stride length as they had minimal information on trunk inclination, i.e., they did not see their trunk inclination as participants training in the third-person perspective did, neither they received information on their truck inclination with concurrent visual feedback. This would back up our idea that perhaps learning the different sub-tasks sequentially might be a better approach than training all sub-tasks simultaneously. However, further research is required to understand participants’ intentions and support this explanation.

In short, we did not find a single combination of visualization perspective and provision of visual feedback that enhances the learning of all sub-tasks simultaneously. We found, however, that the effectiveness of these elements on motor learning might depend on the characteristics of the sub-tasks to be learned [1, 99]. If the goal is to learn the optimal stride length, training participants with a first-person perspective and without feedback seems to be the best combination; while if the aim is to reduce the trunk inclination, training with a third-person perspective and without feedback or with a first-person perspective and with visual feedback seems to result in the best performance.

### Training factors appear to have minimal impact on the user experience

In terms of embodiment, our system showed that a high sense of embodiment over the virtual avatar was achieved independently of the perspective and visual feedback. Although the avatar did not always move as the participant did (e.g., when a step was triggered), this visual and proprioceptive incongruence does not seem to hamper the sense of embodiment, nor any of the subcomponents of embodiment, namely *Body ownership*, *Agency*, and *(Self-)location*. Nonetheless, before a step was triggered, the avatar congruently matched the movements of the participants, probably eliciting the sense of embodiment [100]. Similar levels of embodiment have been observed in previous studies where the incongruence of visual and proprioceptive information had little effect [101]. Thus, our IVR seems to induce high levels of body ownership and agency over the avatar, regardless of the perspective, despite the incongruity between the visual and proprioceptive information once a step was triggered.

The visual feedback did not affect the embodiment. Unexpectedly, the first-person perspective did not result in higher levels of embodiment over the avatar than the third-person perspective, contrary to previous findings [20–22]. We only found a significantly higher reported value of *Self-location* in the first-person perspective vs. third-person perspective.

Concerning cybersickness, relatively low values of the SSQ were reported, with participants feeling no more than a mild general discomfort that ceased as the exposure to the HMD-IVR finished. According to Stanney et al. (1997), simulator scores exceeding 20 on the SSQ are categorized as “bad simulators” [102]. The reported values in our VE range from 16 to 55, with an average score of 29. However, it is important to note that the original study from Stanney et al. focused on military navigators and this threshold may be stringent for our context. In addition, the differences detected by Stanney et al. in the SSQ scores suggested that VE systems produce different symptoms compared to simulator systems [102]. Thus, comparisons between simulator systems and VE systems should be done with caution.

Interestingly, receiving visual feedback significantly increased the *Disorientation* subcomponent scores of the SSQ. Perhaps staying attentive to the visual cues made participants pay less attention to the environment, increasing the feeling of disorientation as participants tried to process and respond to visual information. An analogy to illustrate this is the increase in “sensory mismatch” that occurs when reading in a moving car [103–105]. Based on these findings and taking into account that cybersickness is a common issue in VEs [36, 38, 106], especially for inexperienced users [106], we consider our HMD-IVR-based system safe in terms of cybersickness.

Finally, all the aforementioned aspects define the usability of our system, which was rated as marginally acceptable, according to the scores proposed by Bangor et al. [91]. We consider that the complexity of the task, as well as the limited time that we allowed participants to practice (see Additional file 7), most likely had an impact on this outcome. This is in line with the results from the RTLX questionnaire, which showed a relatively high reported workload, especially during baseline and training. However, it is important to note that the interpretation of the NASA-(R)TLX scores is a current limitation [88]. Addressing this concern, Grier (2015) conducted a comprehensive meta-analysis, defining the range and cumulative frequencies of NASA-TLX scores from over 200 publications [107]. Considering the score reported after the training phase in our study ($56.30 \pm 12.55$), it surpasses the mean value of the RTLX scores documented by Grier ($45.29 \pm 14.99$) and exceeds 60% of the scores obtained from studies using both weighted and unweighted methods. However, when focusing solely on scores derived from domains like video games or robot operation, our value aligns with the midpoint of observed scores (56.60 and 56, respectively). To put our study in the context of more recent literature in the field of IVR, the results of our RTLX questionnaire are similar to those reported by Wenk et al. (2021), who employed a rather complex dual motor-cognitive task using HMDs to evaluate, among others user affects, self-reported levels of cognitive load [11]. In any case, the workload was reduced as the experiment progressed, suggesting that the task became less challenging as the learning advanced.

We also observed that training with visual feedback significantly reduced the *Physical demand* subcomponent of the RTLX from baseline to training to a greater extent than training without visual feedback. Participants may have optimized their movements as a result of the feedback, reducing the physical activity required to complete the task. We also observed that, compared to the third-person perspective, the first-person perspective showed a significant increase in the perceived *Performance* from baseline to training, as well as a significant decrease in the *Effort* subcomponent. Perhaps walking in the first-person perspective may have offered an advantage in task execution and interpretation, given that this training modality aligns with the natural way we walk and observe the world. This alignment could have contributed to a perceived reduction in *Effort*, as opposed to walking in the third-person view. Furthermore, because participants training in the first-person perspective could not externally observe their execution, they might have taken a less stringent approach to their performance.

### Lessons learned and implications for motor learning and gait rehabilitation

In this study, we investigated how different factors in VR impact the motor learning of a complex task relevant to gait rehabilitation. The main lesson learned from this study is that neither the perspective nor the provision of visual feedback appears to make a significant difference in improving the complex task in hand. Instead, based on the observed interaction effects of both factors on motor learning of the different sub-tasks required to master the complex task, it seems that the key is to train each of the sub-tasks independently using a different combination of person’s perspective and visual feedback. Yet, it is unclear if this finding is generalizable. For example, it is possible that an experiment with a simpler visualization, or with more training time, would result in different outcomes. Future research needs to go in that direction and find the most suitable combination of perspective and visual feedback that maximizes the learning of each sub-task in successive order.

Insights from this study may be valuable not only for the general field of motor learning in immersive virtual environments, but also provide relevant insights for the rehabilitation field. Our experimental set-up aimed to replicate conditions in healthy participants that resemble to some extent those experienced by individuals with sensorimotor loss during their initial experience with a wearable lower-limb exoskeleton. We added a balance board to induce instability, simulating the increased challenge and need for compensatory strategies that may be analogous to those experienced by people with a lack of sensorimotor functions. Furthermore, the virtual environment intensified the simulation of lacking sensory information by forcing participants to determine their body configuration through sight, rather than proprioception or touch. This resulted in excessive trunk inclination during training due to the unstable platform, similar to the trunk inclination observed, for example, in people with SCI who over-rely on walkers. Nevertheless, we note that rather than resembling sensory loss, we created a sensory conflict between visual information and the sense of body position (proprioception) and movement (kinesthesia). Therefore, the sensory stimuli experienced by the participants in our study did not fully capture the complexity or absence of sensations encountered by individuals with sensory loss. Nevertheless, we believe that a platform similar to the one described in this study could serve as a training simulator. This tool could help exoskeleton developers understand some of the challenges faced by people with sensorimotor loss when using an exoskeleton and allow for fast and modifiable research in a realistic environment. In the future, we aim to improve the system and use it to potentiate the gait rehabilitation process in people with sensorimotor disorders, such as SCI.

## Study limitations

The present study suffers from several limitations. First, the small sample size (40 participants, 10 per condition) may have prevented us from achieving statistical significance. Likewise, we did not perform the study with people with sensorimotor disorders but with healthy young adults, which limits the extent to which our findings can be generalized to the final target population. Second, the time assigned for practice could have been insufficient for participants to adapt to the system and fully comprehend the dynamics of the task, potentially limiting the system’s ability to enhance learning. Third, we only tested for short-term retention right after the training phase, and thus, we cannot infer conclusions regarding long-term learning. Finally, we acknowledge that our study design was rather complex, with several conditions and hypotheses. Future research could benefit from investigating more focused experimental designs.

## Conclusion

We developed an HMD-IVR-based system to investigate the efficacy of IVR in facilitating motor learning, specifically focusing on motor complex tasks such as learning how to use a wearable lower-limb exoskeleton for overground walking. We examined the system in healthy participants under conditions that simulate the lack of motor control and proprioception of the lower limbs that people with sensory loss experience in real life. Through this experiment, we aimed to investigate the effect of first- vs. third-person perspectives and concurrent visual feedback on enhancing motor learning of this particularly complex task. Our findings suggest that the system allowed for learning the virtual walking task. However, we did not find a combination of a person’s perspective and visual feedback that effectively improves all required skills to perform this especially complex task successfully. Instead, it appears that the key lies in the correct selection of a person’s perspective and visual feedback based on each sub-task characteristics that make up the virtual walking task. Future research needs to go in that direction and find the most suitable combination of perspective and visual feedback that maximizes the learning of each sub-task in consecutive order [66–107].

## Supplementary information

Additional file 1.

Additional file 2.

Additional file 3.

Additional file 4.

Additional file 5.

Additional file 6.

Additional file 7.

## Data Availability

The Unity project that contains the full experiment described in this manuscript and the results data for the full experiment can be found in: https://github.com/tud-hri/ExoskeletonLearningExperiment_public

## Abbreviations

ANOVA: Analysis of variance
B: Baseline test
BH: Body height
HMD: Head-mounted displays
HMD-IVR: Head-mounted displays in an immersive virtual reality (HMD-IVR)
IK: Inverse kinematics
IMU: Inertial measurement unit
IVR: Immersive virtual reality
LMM: Linear mixed-effects model
NF: No feedback - First perspective
NT: No feedback - Third perspective
R: Retention test
SCI: Spinal cord injury
SHCs: Secondary health conditions
SSQ: Simulator sickness questionnaire
SUS: System usability scale
T: Training
TS: Total score
RTLX: Raw task load index
VE: Virtual environments
VF: Yes feedback - First perspective
VR: Virtual Reality
VT: Yes feedback - Third perspective
1PP: First person-perspective
3PP: Third person-perspective
