# Supplementary material for: Immersive virtual reality for learning exoskeleton-like virtual walking: a feasibility study
Source: J Neuroeng Rehabil. 2024 Nov 1;21:195. doi: 10.1186/s12984-024-01482-y (PMC11531127; doi:10.1186/s12984-024-01482-y)
Supplement: Supplementary file 2 — Additional file 2. [file 12984_2024_1482_MOESM2_ESM.pdf]

Virtual Reality-Based Gait Training

A First Step Towards Accelerate the Learning of Using an Exoskeleton

Inability to stand and/or walk is one of the major consequences of **SPINAL CORD INJURY,** leading to **wheelchair dependency**

WEARABLE EXOSKELETONS make possible to assist gait (walking) in people with spinal cord injury to recover independent stand and walk

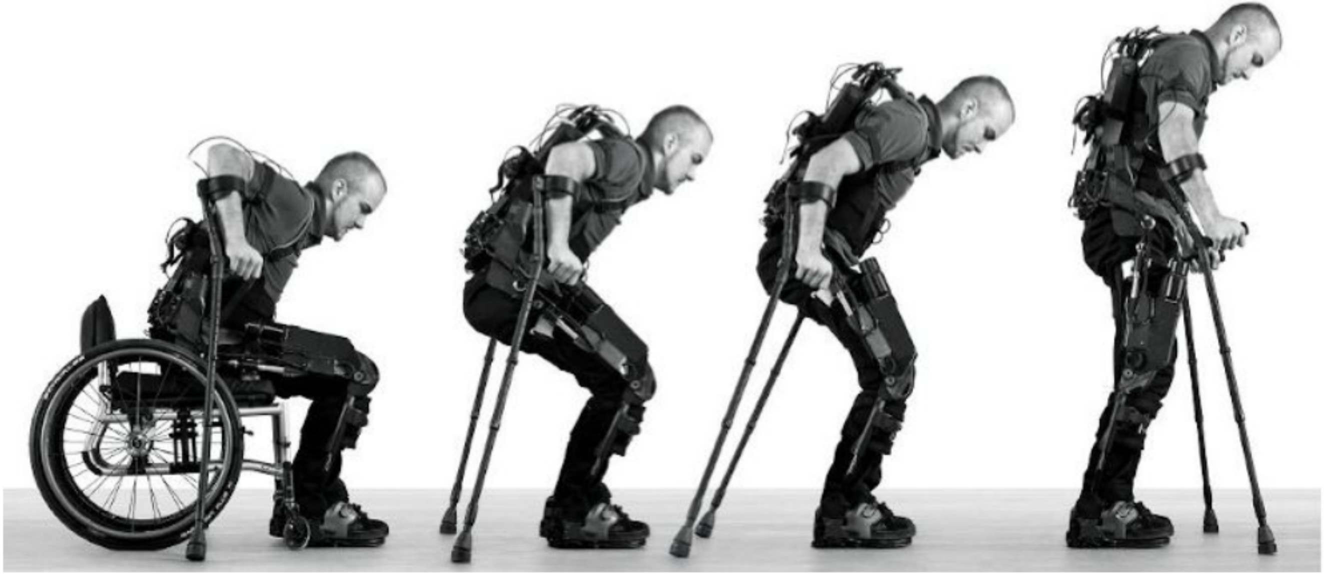

To control a robotic exoskeleton, sensing the state of the patient’s body during walking is a requirement

+

People with spinal cord injury not only lose the ability to control their muscles, but also sensory information

=

Learning to use an exoskeleton is **time-consuming** and requires a lot of **effort** by the user

Example of a patient learning to use a wearable exoskeleton:

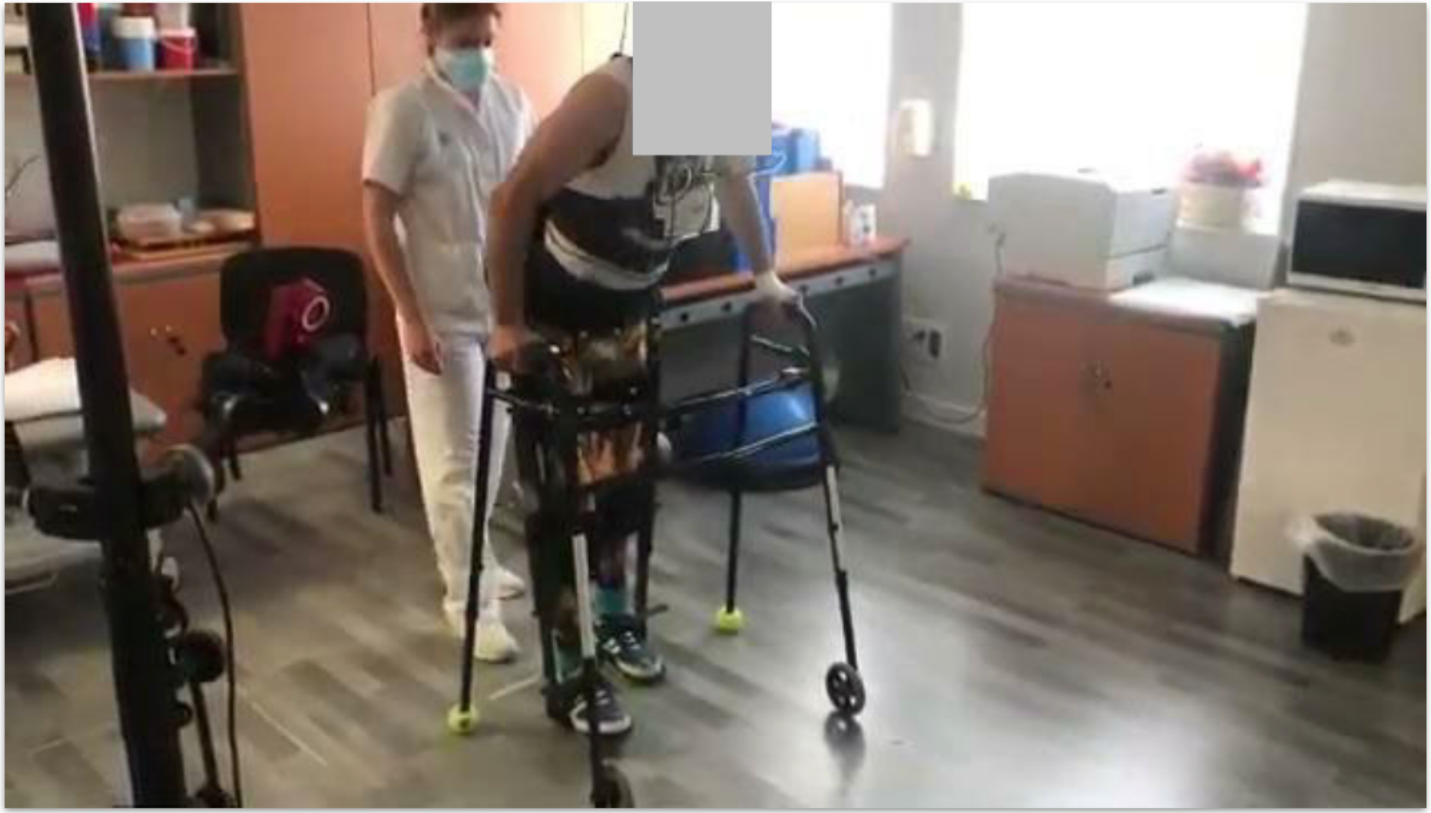

MOST COMMON PROBLEMS FOR PATIENTS WHEN USING A WEARABLE EXOSKELETON

They look at their feet all the time → There is not another to way to know where they are

THEY DON'T FEEL THE LEGS!

Put too much weight on the walker → The walker is their balance support

THEY CAN'T MOVE THEIR LEG MUSCLES!

They have to learn a completely new way to walk

LEARNING TO USE A NEW DEVICE + ADAPTING TO THEIR "NEW BODY"

In this study, you are going to feel (a bit) the problems that patients face when learning to use an exoskeleton

SET-UP:

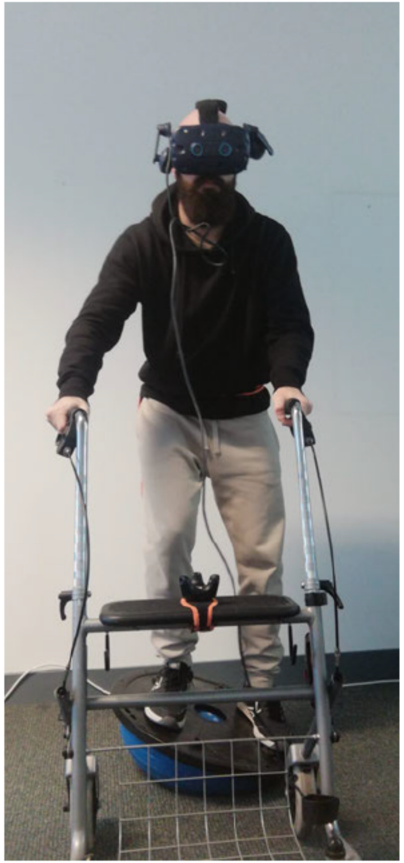

You are going to control a virtual avatar and a walker with your own movements

while

learning to use a virtual wearable exoskeleton

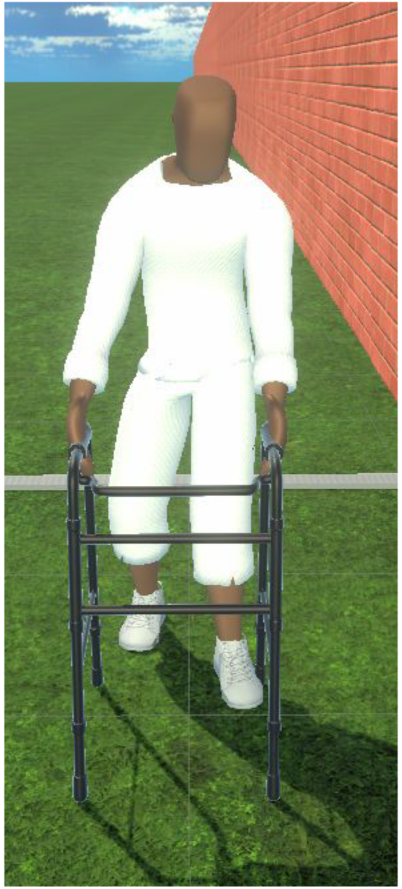

SET-UP:

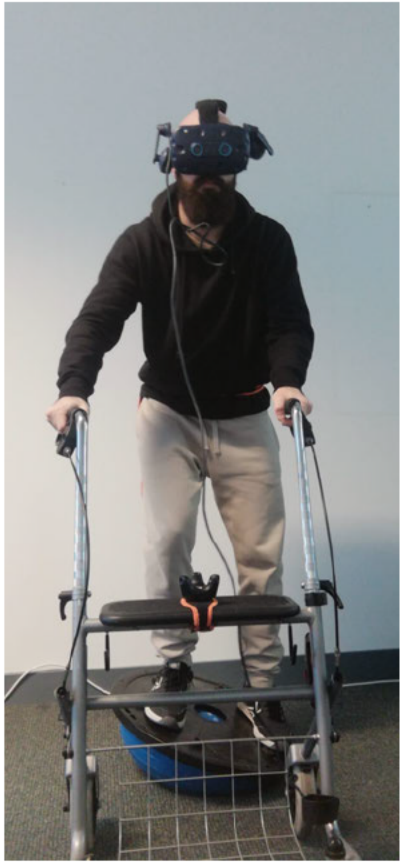

Why am I learning to use a device intended for walking while I'm standing?

We want your experience to be similar to that of the patients. Therefore, we want to remove the proprioception (sense of feeling the position of your body without looking) from your legs. You are not walking, but the virtual avatar does! If you want to see the movement of your legs you should look at the virtual avatar

Why am I using a balance board?

We want to make the experience as close as possible to that of a real patient. The balance board adds extra imbalance. Remember: one of the main problems in people with spinal cord injury is the difficulty for balancing!

PROTOCOL:

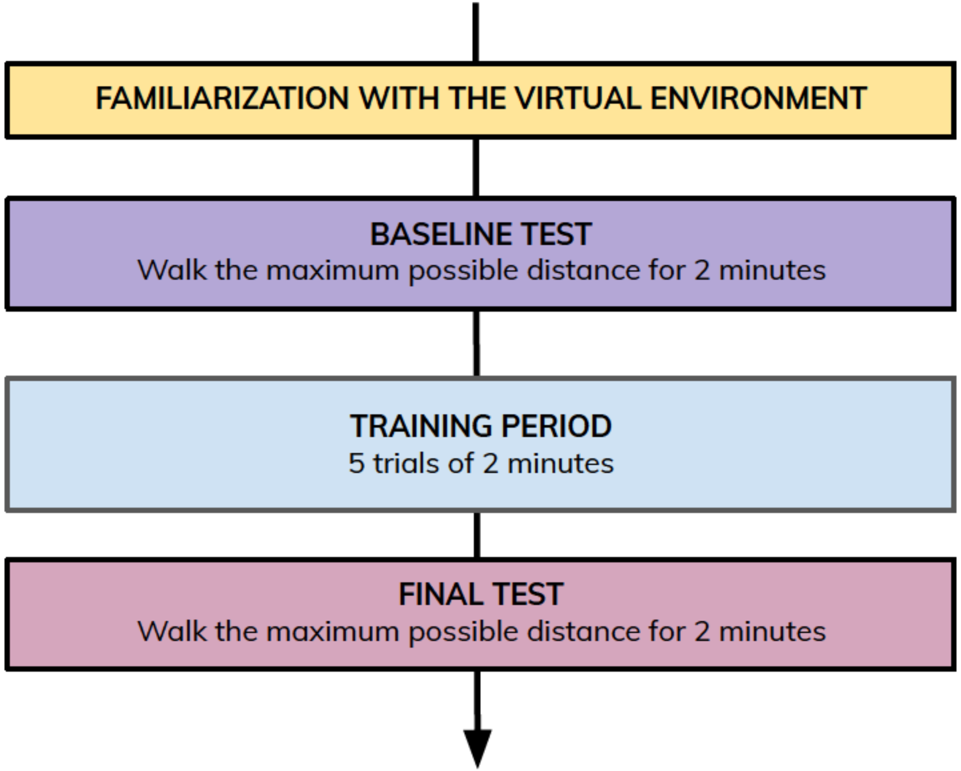
FAMILIARIZATION WITH THE VIRTUAL ENVIRONMENT
BASELINE TEST
Walk the maximum possible distance for 2 minutes
TRAINING PERIOD
5 trials of 2 minutes
FINAL TEST
Walk the maximum possible distance for 2 minutes

How to control the virtual exoskeleton:

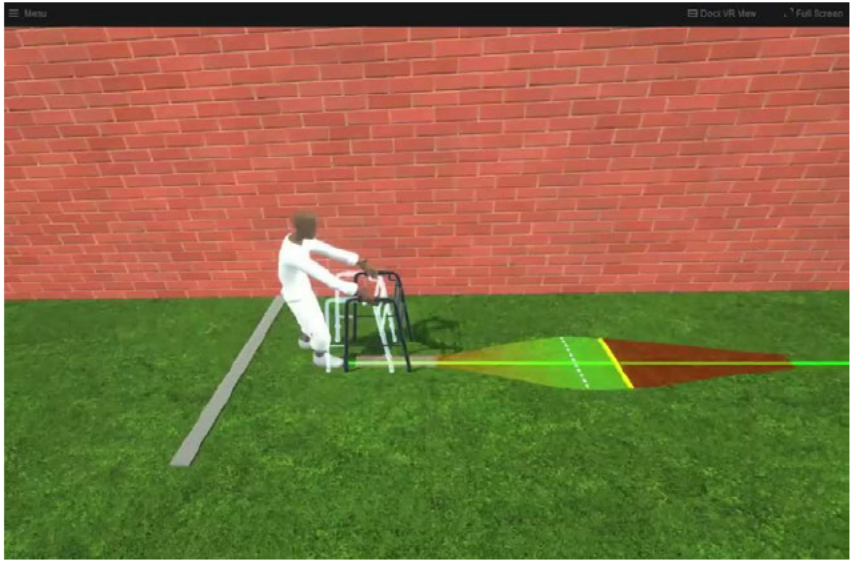

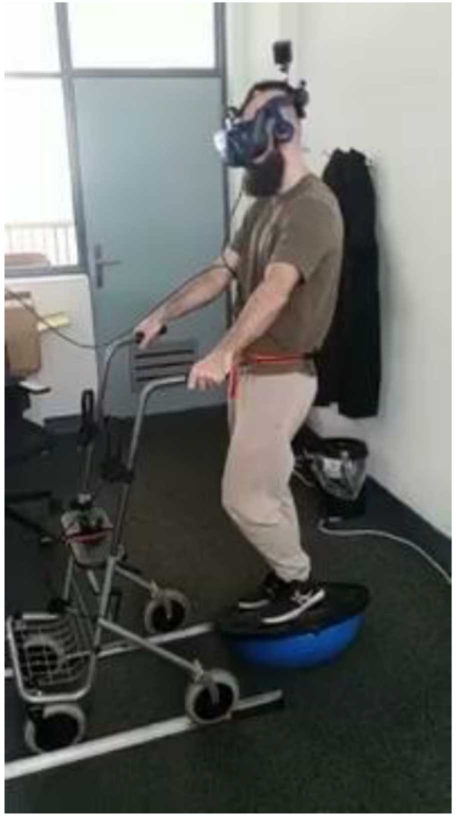

Rules to control the virtual exoskeleton:

1. Move the walker forward: You need some space to move the leg forward

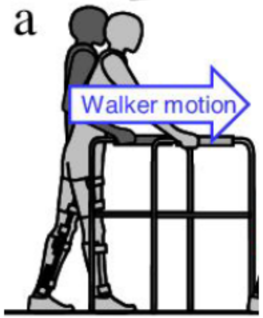

2. Weight shifting: You must put your body weight on your leading leg. Otherwise, how are you going to be able to raise the stepping leg to cross forward?

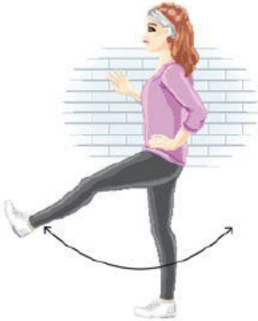

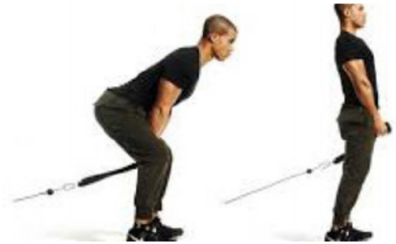

3. Hip thrust: How could I move my leg forward if I can't move my leg? MOVE YOUR HIP!* Imagine your leg as a pendulum

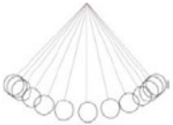

* The faster the movement, the longer the step

Extra Tip

After each step, move the walker backward

If you see the green walker:

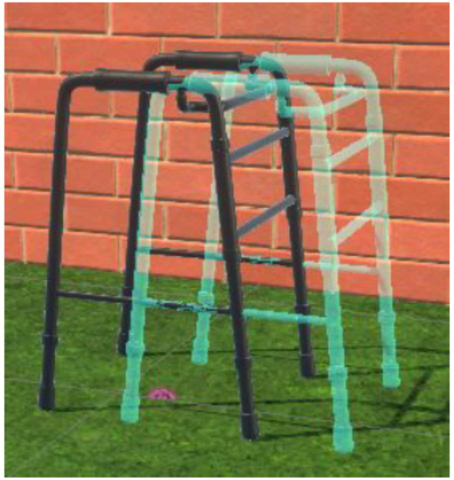

Move it backward until you match the position with the black walker

Only after this, you can start another step

One last thing!

If your step was **too long** that you would collide with the walker…

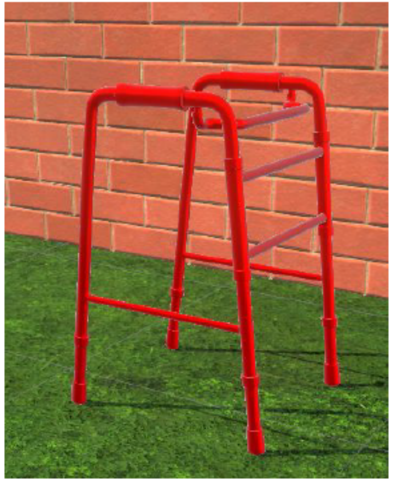

… the walker would turn red and that step will **not** be valid

and remember... **DON'T CHEAT**

Hold the walker properly

GOOD

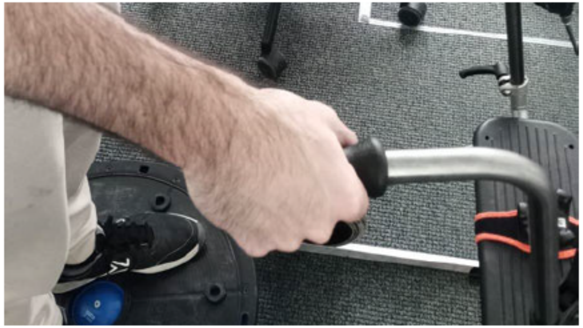

BAD

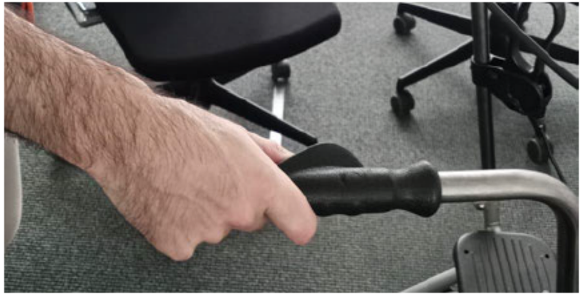

Don't jump

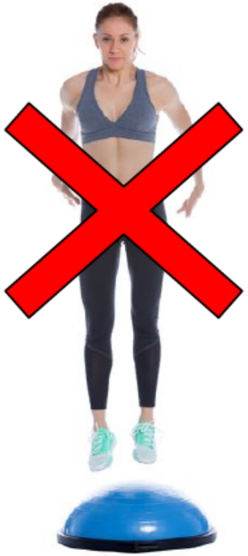

Example of what you should do:

⚠ The step will be triggered only if you do the movement properly!

NO STEP IF there is not enough (or too much) **weight shifting**

Your weight should be balanced over your leading/front foot

NO STEP IF you don't reach the minimum acceleration threshold with your **hip thrust**

NO STEP IF your **hip thrust** movement is too small

During your acceleration, you should move at least 2 cm forward

NO STEP IF possible **collision** with the walker

MAIN GOAL:

Stay upright! Don’t tilt your trunk and rely on the walker as little as possible

Be efficient with your step length Not too short, not too long

Don’t collide with the walker Be sure that the space between you and the walker is enough for the step length

Walk the maximum distance possible

Example in real life:

WRONG MOVEMENT

GOOD MOVEMENT

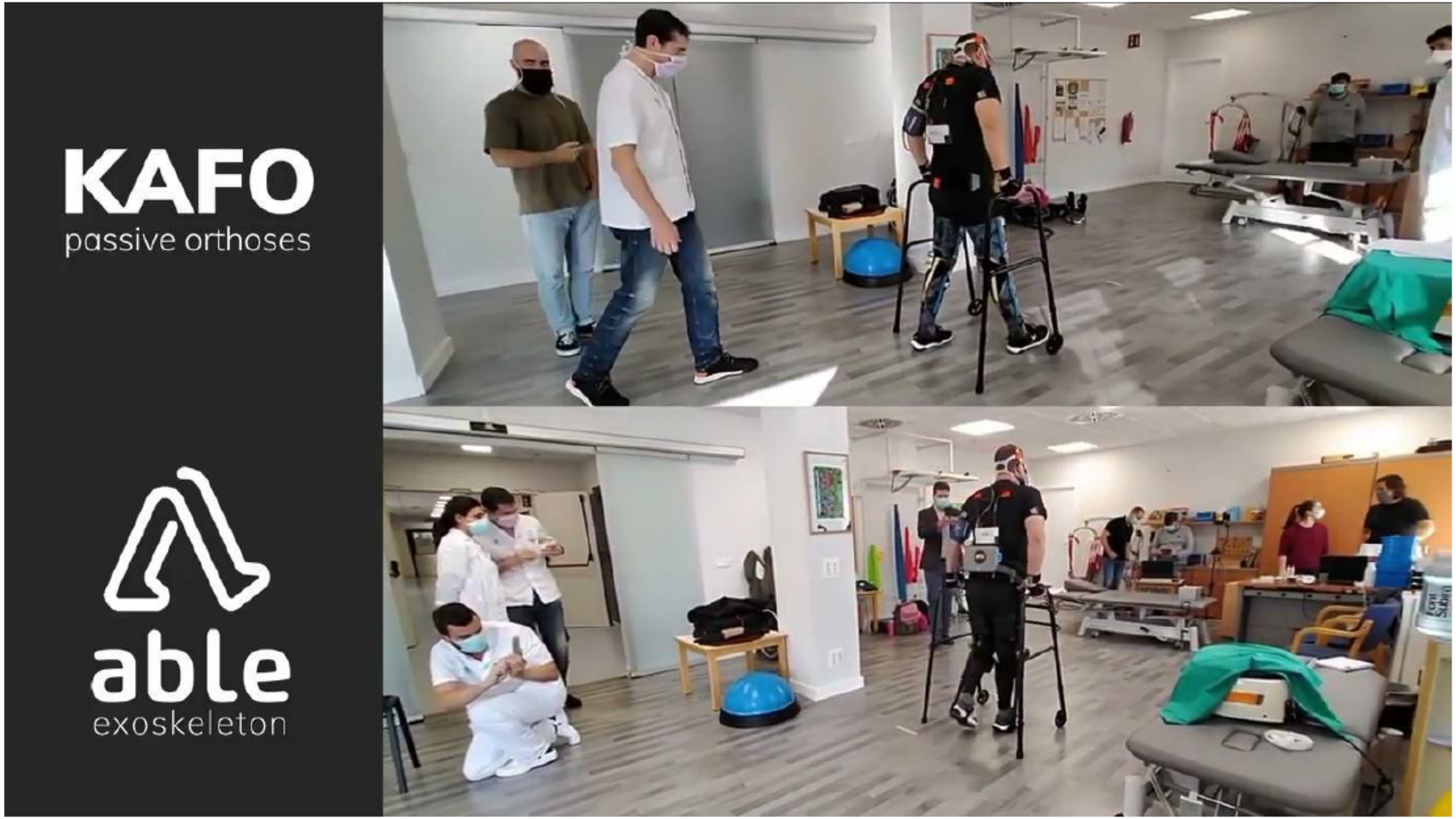

NOW IT’S YOUR TURN!

Any question?
