# Supplementary material for: Immersive virtual reality for learning exoskeleton-like virtual walking: a feasibility study
Source: J Neuroeng Rehabil. 2024 Nov 1;21:195. doi: 10.1186/s12984-024-01482-y (PMC11531127; doi:10.1186/s12984-024-01482-y)
Supplement: Supplementary file 3 — Additional file 3. [file 12984_2024_1482_MOESM3_ESM.pdf]

Virtual Reality-Based Gait Training

A First Step Towards Accelerate the Learning of Using an Exoskeleton

User Manual for the Visual Feedback

All the feedback you need is in this fusiform object:

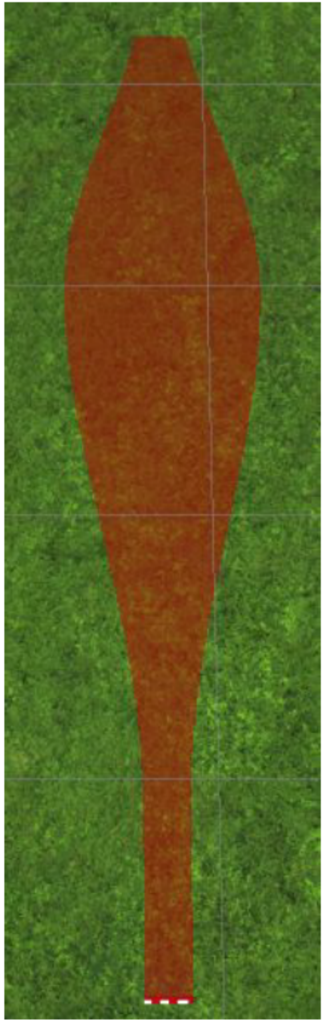

Object lateral position Controlled by your HIP POSITION

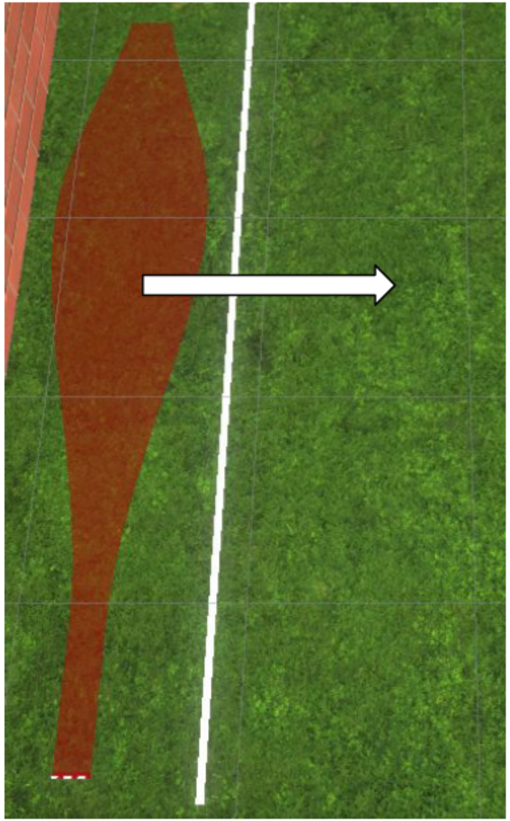

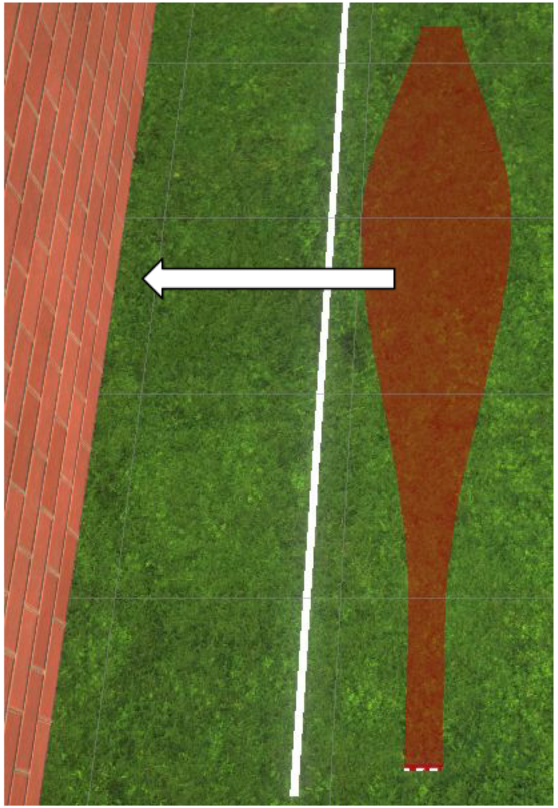

Object length Controlled by your TRUNK INCLINATION

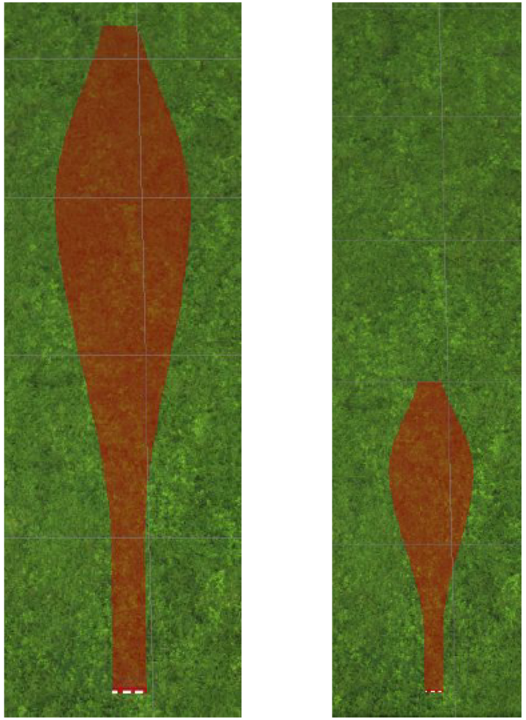

When there is no trunk inclination → The object length is maximum (and vice versa)

Weight Shifting Shown when the object touches the white line in front of your leg

Move your hip until the white line turns green

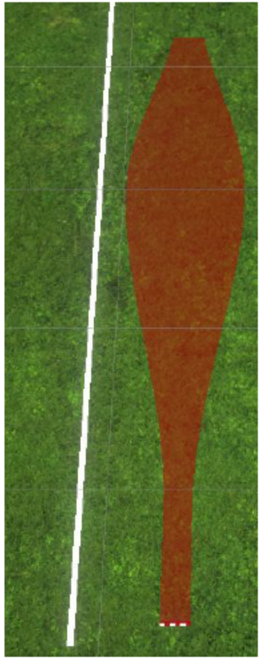

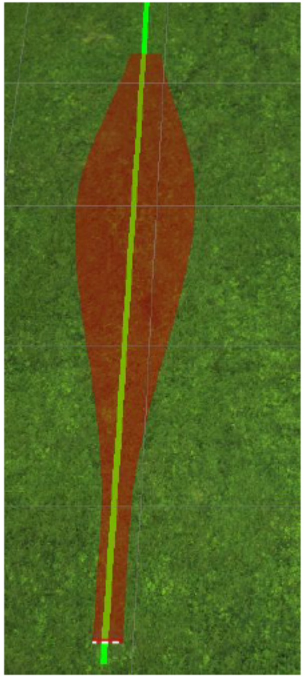

Don’t do the hip thrust until you see the green line

Hip Thrust Shown when the object changes the color

Maximum hip acceleration
=
Maximum step length

Previous acceleration
=
Previous step length

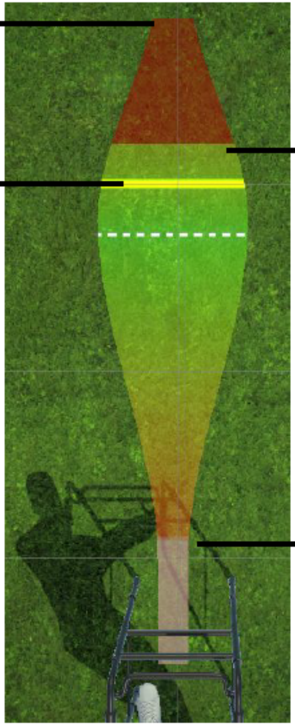

Current acceleration
=
Current step length

Minimum threshold to trigger the step

The faster the hip thrust → The higher it goes → The longer the step length

Walker position Shown with the intersection where colors change

Walker position

If your acceleration goes above this value, it counts as a walker collision

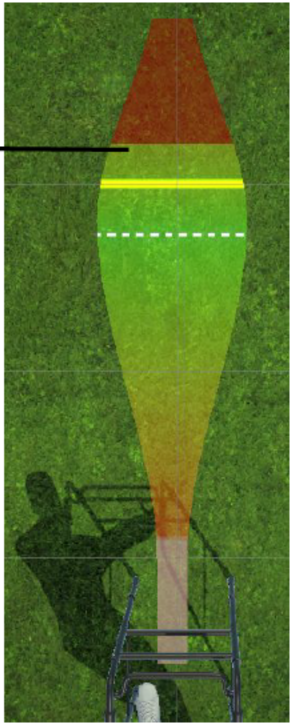

The position of the walker may limit your step length

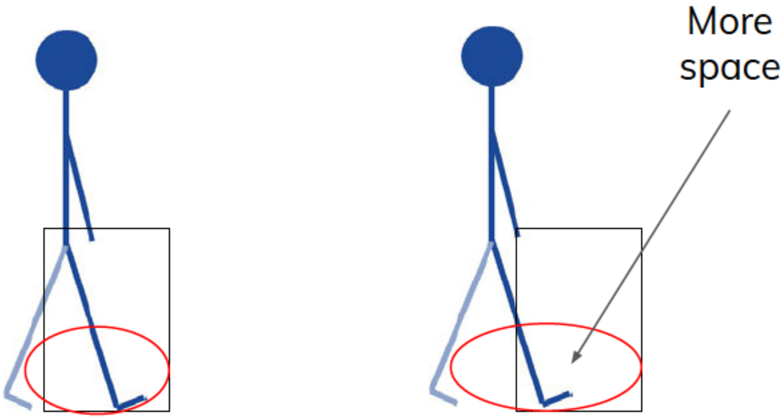

The closer the walker, the smaller space you have to do a step

Some extra feedback:

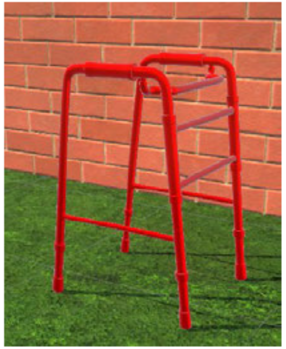

❗ If you **collide with the walker**, the walker turns **red** and that step is not valid. TRY AGAIN

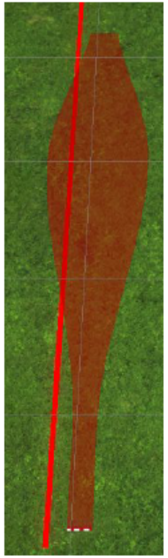

❗ If you started the hip thrust movement out of the weight shifting zone, the white line in front of you turns **red**, and no step is done. DO THE WEIGHT SHIFT AND TRY AGAIN

Game Score

During the **training period** you will receive a score per each step based on its performance

Game Score

When the object length is maximum, so is the score.

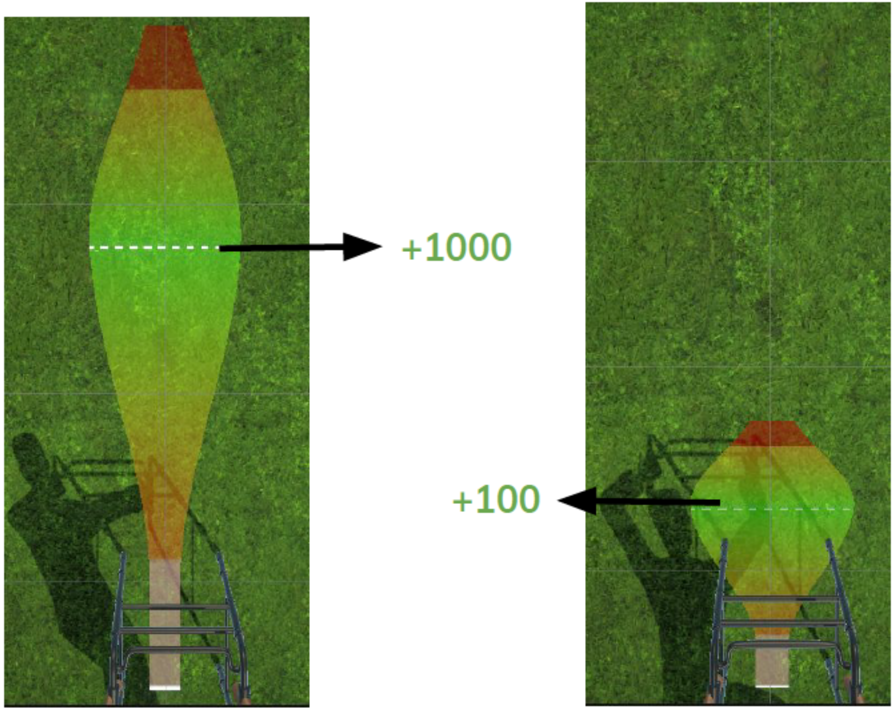

Stay as upright as possible to get more points!

Game Score

Each person has an optimal step length, which depends on the previous step

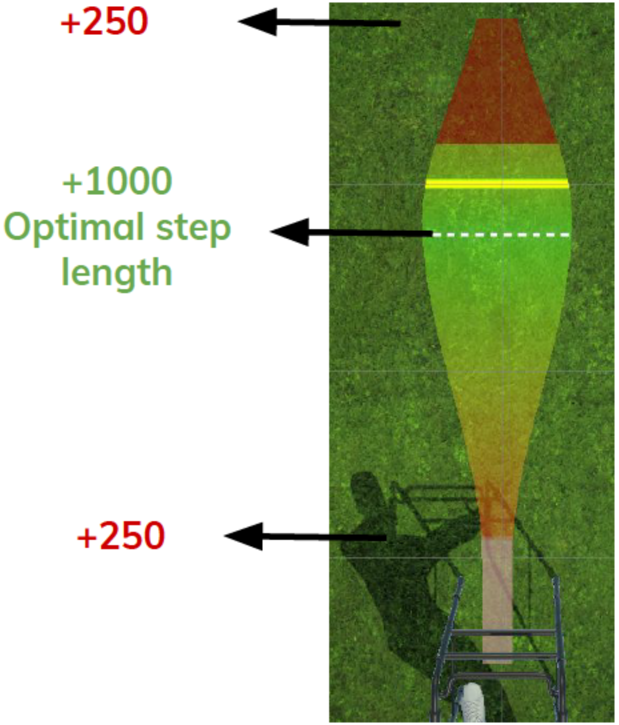

The optimal step length is shown with a white dashed line, in green, and in the widest part of the object

Try to do as many optimal steps as possible to get more points!

Game Score

The more **distance** you walk, the higher score you will get

More steps=more points... or something
