# Supplementary material for: Immersive virtual reality for learning exoskeleton-like virtual walking: a feasibility study
Source: J Neuroeng Rehabil. 2024 Nov 1;21:195. doi: 10.1186/s12984-024-01482-y (PMC11531127; doi:10.1186/s12984-024-01482-y)
Supplement: Supplementary file 4 — Additional file 4. [file 12984_2024_1482_MOESM4_ESM.pdf]

Supplementary Table 1 Components and questions from the Virtual Embodiment questionnaire* with the weightings used to compute the overall embodiment score. Participants responded on a Likert scale between 1 and 7 points (1 indicated "Strongly disagree" and 7 indicated "Strongly agree").

#	Component	Weight	Question
Q1	Body Ownership	1/3	It seemed like the virtual body was my body
Q2	Body Ownership	1/3	It seemed like the virtual body parts were my body parts
Q3	Body Ownership	1/3	It seemed like I was looking directly at my own body
Q4	Location	1	It seemed like my body was in the location where the virtual body was
Q5	Agency	1/2	It seemed like I was in control of the virtual body
Q6	Agency	1/2	It seemed like I was causing the movements I saw

* Wenk, N. et al. Effect of immersive visualization technologies on cognitive load, motivation, usability, and embodiment. Virtual Real. DOI: 10.1007/S10055-021-00565-8 (2021).

Supplementary Table 2 Components and questions from the System Usability Scale (SUS)*. Participants had five response options on a Likert scale, from 1-"Strongly disagree" to 5-"Strongly agree". The total score is the average score from all the questions.

#	Question
Q1	I think that I would like to use this system frequently
Q2	I found the system unnecessarily complex
Q3	I thought the system was easy to use
Q4	I think that I would need the support of a technical person to be able to use this system
Q5	I found the various functions in this system were well integrated
Q6	I thought there was too much inconsistency in this system
Q7	I would imagine that most people would learn to use this system very quickly
Q8	I found the system very cumbersome to use
Q9	I felt very confident using the system
Q10	I needed to learn a lot of things before I could get going with this system

* Brooke, J. SUS: A 'Quick and Dirty' Usability Scale (CRC Press, 1996).

Supplementary Table 3 Symptoms and categories for the Simulation Sickness Questionnaire (SSQ)*. Participants provided subjective severity ratings of the symptoms on a four-point scale (None = 0, Slight = 1, Moderate = 2, Severe = 3). Nausea Score = [A] · 9.54; Oculomotor Score = [B] · 7.58; Disorientation Score = [C] · 13.92; Total Score = ([A] + [B] + [C]) · 3.74.

#	SSQ Symptom	Nausea	Oculomotor	Disorientation
Q1	General discomfort	○	○	
Q2	Fatigue		○	
Q3	Headache		○	
Q4	Eyestrain		○	
Q5	Difficulty focusing		○	○
Q6	Increased salivation	○		
Q7	Sweating	○		
Q8	Nausea	○		○
Q9	Difficulty concentrating	○	○	
Q10	Fullness of the Head			○
Q11	Blurred vision		○	○
Q12	Dizziness with eyes open			○
Q13	Dizziness with eyes closed			○
Q14	Vertigo			○
Q15	Stomach awareness	○		
Q16	Burping	○		
		[A]	[B]	[C]

* Kennedy, R. S., Lane, N. E., Berbaum, K. S. & Lilienthal, M. G. Simulator Sickness Questionnaire: An enhanced method for quantifying simulator sickness. The Int. J. Aviat. Psychol. 3, 203–220, DOI: 10.1207/S15327108IJAP0303_3 (1993).

Supplementary Table 4 Questions from the Raw Task Load Index (RTLX)*. Each question was answered using a markless slider on a scale of 21 marks. The score of each subscale consists of the number of lines the participant marked subtracted by 1, and multiply by 5 (to have a range of values from 0 to 100). The total score is the mean value from all the subscales.

#	Subscale	Endpoints	Description
Q1	Mental Demand	Low/High	How much mental and perceptual activity was required (e.g., thinking, deciding, calculating, remembering, looking, searching, etc.)? Was the task easy or demanding, simple or complex, exacting or forgiving?
Q2	Physical Demand	Low/High	How much physical activity was required (e.g., pushing, pulling, turning, controlling, activating, etc.)? Was the task easy or demanding, slow or brisk, slack or strenuous, restful or laborious?
Q3	Temporal Demand	Low/High	How much time pressure did you feel due to the rate or pace at which the tasks or task elements occurred? Was the pace slow and leisurely or rapid and frantic?
Q4	Performance	Poor/Good [a]	How successful do you think you were in accomplishing the goals of the task set by the experimenter (or yourself)? How satisfied were you with your performance in accomplishing these goals?
Q5	Effort	Low/High	How hard did you have to work (mentally and physically) to accomplish your level of performance?
Q6	Frustration Level	Low/High	How insecure, discouraged, irritated, stressed, and annoyed versus secure, gratified, content, relaxed, and complacent did you feel during the task?

[a] The Performance subscale used was inverted by mistake (the conventional endpoints are Good/Poor). The reported Performance values were, therefore, reversed during the analysis

* Hart, S. G. Nasa-Task Load Index (NASA-TLX); 20 Years Later:. Proc. Hum. Factors Ergonomics Soc. Annu. Meet. 50, 904–908, DOI: 10.1177/154193120605000909 (2006).
