# Supplementary material for: Immersive virtual reality for learning exoskeleton-like virtual walking: a feasibility study
Source: J Neuroeng Rehabil. 2024 Nov 1;21:195. doi: 10.1186/s12984-024-01482-y (PMC11531127; doi:10.1186/s12984-024-01482-y)
Supplement: Supplementary file 5 — Additional file 5. [file 12984_2024_1482_MOESM5_ESM.pdf]

Supplementary Table 5 Posthoc analysis on the performance change from baseline to retention of the gait metrics. Bold highlighting indicates statistical significance (*** $p < 0.001$, ** $p < 0.01$, * $p < 0.05$). Italic highlighting indicates tendency ($^{\bullet}$ $p < 0.1$)

Gait Metric	Comparison	Mean difference	95% CI	p-value
Trunk inclination [a]	NF vs. YF	5.17	[-4.18, 14.52]	0.454
	YT vs. YF	4.18	[-5.17, 13.53]	0.628
	NT vs. YF	-1.01	[-10.36, 8.34]	0.991
	YT vs. NF	-0.99	[-10.34, 8.36]	0.992
	NT vs. NF	-6.18	[-15.53, 3.17]	0.299
	NT vs. YT	-5.19	[-14.54, 4.16]	0.450
Deviation from the target stride length [a]	NF vs. YF	-13.73	[-26.57, -0.89]	**0.032 ***
	YT vs. YF	-0.79	[-13.96, 12.39]	0.998
	NT vs. YF	2.56	[-10.28, 15.40]	0.949
	YT vs. NF	12.94	[0.10, 25.78]	**0.048 ***
	NT vs. NF	16.29	[-3.79, 28.78]	**0.007 ****
	NT vs. YT	3.34	[-9.50, 16.18]	0.895

NF: NO feedback - 1PP ; YF: YES feedback - 1PP; NT: NO feedback - 3PP; YT: YES feedback - 3PP

[a] Posthoc pairwise comparisons with Tukey corrections
