# Supplementary material for: Immersive virtual reality for learning exoskeleton-like virtual walking: a feasibility study
Source: J Neuroeng Rehabil. 2024 Nov 1;21:195. doi: 10.1186/s12984-024-01482-y (PMC11531127; doi:10.1186/s12984-024-01482-y)
Supplement: Supplementary file 6 — Additional file 6. [file 12984_2024_1482_MOESM6_ESM.pdf]

Supplementary Table 6: Results from the linear mixed-effects model (LMM) statistical analysis of the RTLX questionnaire. Bold highlighting indicates statistical significance (*** $p < 0.001$, ** $p < 0.01$, * $p < 0.05$). Italic highlighting indicates tendency (· $p < 0.1$)

RTLX	Effect	Significance	Model effect size
Total Score	Feedback	b = -0.52 (95% CI: -2.64, 1.61), $t(36)$ = -0.47, p = 0.643	1.10
	Perspective	b = -1.38 (95% CI: -3.51, 0.74), $t(36)$ = -1.25, p = 0.218	
	Time (Training)	**b = -3.00 (95% CI: -4.60, -1.40), $t(72)$ = -3.54, p ¡ 0.001 *****	
	Time (Retention)	**b = -2.67 (95% CI: -4.27, -1.06), $t(72)$ = -3.14, p = 0.002 ****	
	Feedback * Perspective	b = -0.37 (95% CI: -3.37, 2.64), $t(36)$ = -0.23, p = 0.816	
	Feedback * Training	*b = 2.02 (95% CI: -0.25, 4.29), $t(72)$ = 1.68, p = 0.097* ·	
	Feedback * Retention	b = -0.28 (95% CI: -2.55, 1.99), $t(72)$ = -0.24, p = 0.814	
	Perspective * Training	b = 1.68 (95% CI: -0.59, 3.95), $t(72)$ = 1.40, p = 0.165	
	Perspective * Retention	b = -0.08 (95% CI: -2.35, 2.19), $t(72)$ = -0.07, p = 0.945	
	Feedback * Perspective * Training	b = -0.91 (95% CI: -4.12, 2.29), $t(72)$ = -0.54, p = 0.591	
	Feedback * Perspective * Retention	b = 2.45 (95% CI: -0.76, 5.66), $t(72)$ = 1.44, p = 0.153	
Mental demand	Feedback	b = -0.50 (95% CI: -3.67, 2.68), $t(36)$ = -0.30, p = 0.764	0.83
	Perspective	b = 0.00 (95% CI: -3.18, 3.18), $t(36)$ = 0.00, p = 1.000	
	Time (Training)	b = -1.20 (95% CI: -3.63, 1.23), $t(72)$ = -0.93, p = 0.354	
	Time (Retention)	b = -1.90 (95% CI: -4.33, 0.53), $t(72)$ = -1.47, p = 0.144	
	Feedback * Perspective	b = 0.50 (95% CI: -3.99, 4.99), $t(36)$ = 0.21, p = 0.832	
	Feedback * Training	b = 1.20 (95% CI: 2.24, 4.64), $t(72)$ = 0.66, p = 0.512	
	Feedback * Retention	b = 0.60 (95% CI: -2.84, 4.04), $t(72)$ = 0.33, p = 0.743	
	Perspective * Training	b = 0.10 (95% CI: -3.34, 3.54), $t(72)$ = 0.05, p = 0.956	

Supplementary Table 6 – continued from previous page

RTLX	Effect	Significance	Model effect size
	Perspective * Retention	b = -2.40 (95% CI: -5.84, 1.04), $t(72)$ = -1.32, p = 0.191	
	Feedback * Perspective * Training	b = -0.30 (95% CI: -5.16, 4.56), $t(72)$ = -0.12, p = 0.908	
	Feedback * Perspective * Retention	b = 2.70 (95% CI: -2.16, 7.56), $t(72)$ = 1.05, p = 0.297	
	Feedback	b = -0.60 (95% CI: -3.31, 2.11), $t(36)$ = -0.42, p = 0.672	
	Perspective	b = -0.3 (95% CI: -3.01, 2.41), $t(36)$ = -0.21, p = 0.832	
	Time (Training)	**b = -3.20 (95% CI: -5.13, -1.27), $t(72)$ = -3.13, p = 0.003 ****	
	Time (Retention)	*b = -2.00 (95% CI: -3.93, -0.07), $t(72)$ = -1.96, p = 0.054 ·*	
	Feedback * Perspective	b = -0.70 (95% CI: -4.53, 3.13), $t(36)$ = 0.35, p = 0.727	
Physical demand	Feedback * Training	**b = 3.90 (95% CI: 1.17, 6.63), $t(72)$ = 2.70, p = 0.009 ****	1.16
	Feedback * Retention	b = 0.60 (95% CI: -2.13, 3.33), $t(72)$ = 0.41, p = 0.679	
	Perspective * Training	b = 0.50 (95% CI: -2.23, 3.23), $t(72)$ = 0.35, p = 0.731	
	Perspective * Retention	b = -0.70 (95% CI: -3.43, 2.03), $t(72)$ = -0.48, p = 0.630	
	Feedback * Perspective * Training	b = -1.40 (95% CI: -5.27, 2.47), $t(72)$ = -0.68, p = 0.496	
	Feedback * Perspective * Retention	b = 1.60 (95% CI: -2.27, 5.47), $t(72)$ = 0.78, p = 0.437	
	Feedback	b = -0.30 (95% CI: -3.53, 2.93), $t(36)$ = -0.18, p = 0.859	
	Perspective	b = -2.00 (95% CI: -5.23, 1.23), $t(36)$ = -1.19, p = 0.242	
	Time (Training)	b = -1.70 (95% CI: -4.14, 0.74), $t(72)$ = -1.32, p = 0.191	
	Time (Retention)	b = -1.00 (95% CI: -3.43, 1.44), $t(72)$ = -0.78, p = 0.440	
	Feedback * Perspective	b = -0.80 (95% CI: -5.37, 3.77), $t(36)$ = -0.33, p = 0.738	
Temporal demand	Feedback * Training	b = 0.60 (95% CI: -2.85, 4.05), $t(72)$ = 0.33, p = 0.743	0.86
	Feedback * Retention	b = -0.60 (95% CI: -4.05, 2.85), $t(72)$ = -0.33, p = 0.743	

Supplementary Table 6 – continued from previous page

RTLX	Effect	Significance	Model effect size
	Perspective * Training	$b = 0.50$ (95% CI: -2.95, 3.95), $t(72) = 0.27$, $p = 0.785$	
	Perspective * Retention	$b = 0.50$ (95% CI: -2.94, 3.95), $t(72) = 0.27$, $p = 0.785$	
	Feedback * Perspective * Training	$b = 1.80$ (95% CI: -3.07, 6.67), $t(72) = 0.70$, $p = 0.487$	
	Feedback * Perspective * Retention	_$b = 4.40$ (95% CI: -0.47, 9.27), $t(72) = 1.71$, $p = 0.092$_ ·	
Performance	Feedback	$b = 0.30$ (95% CI: -3.04, 3.64), $t(36) = 0.17$, $p = 0.864$	1.00
	Perspective	$b = -1.70$ (95% CI: -5.04, 1.64), $t(36) = -0.98$, $p = 0.334$	
	Time (Training)	**$b = -6.70$ (95% CI: -9.46, -3.94), $t(72) = -4.59$, p ¡ 0.001 *** **	
	Time (Retention)	**$b = -4.90$ (95% CI: -7.66, -2.14), $t(72) = -3.36$, $p = 0.001$ ****	
	Feedback * Perspective	$b = -0.90$ (95% CI: -5.63, 3.83), $t(36) = -0.37$, $p = 0.716$	
	Feedback * Training	$b = 3.10$ (95% CI: -0.80, 7.00), $t(72) = 1.50$, $p = 0.137$	
	Feedback * Retention	$b = -2.20$ (95% CI: -6.10, 1.70), $t(72) = -1.07$, $p = 0.230$	
	Perspective * Training	**$b = 4.90$ (95% CI: 1.00, 8.80), $t(72) = 2.38$, $p = 0.020$ ***	
	Perspective * Retention	$b = 0.50$ (95% CI: -3.40, 4.40), $t(72) = 0.24$, $p = 0.809$	
	Feedback * Perspective * Training	$b = -3.80$ (95% CI: -9.32, 1.72), $t(72) = -1.30$, $p = 0.197$	
	Feedback * Perspective * Retention	$b = 3.50$ (95% CI: -2.02, 9.02), $t(72) = 1.20$, $p = 0.234$	
Effort	Feedback	_$b = -2.50$ (95% CI: -5.00, 0.01), $t(36) = -1.92$, $p = 0.063$_ ·	1.06
	Perspective	**$b = -2.80$ (95% CI: -5.31, -0.29), $t(36) = -2.14$, $p = 0.038$ ***	
	Time (Training)	**$b = -3.40$ (95% CI: -5.23, -1.57), $t(72) = -3.51$, p¡ 0.001 *****	
	Time (Retention)	**$b = -3.20$ (95% CI: -5.03, 1.37), $t(72) = -3.30$, $p = 0.002$ ****	
	Feedback * Perspective	$b = 2.00$ (95% CI: -1.54, 5.54), $t(36) = 1.09$, $p = 0.285$	
	Feedback * Training	_$b = 2.60$ (95% CI: 0.01, 5.19), $t(72) = 1.90$, $p = 0.062$_ ·	

Supplementary Table 6 – continued from previous page

RTLX	Effect	Significance	Model effect size
	Feedback * Retention	b = 1.70 (95% CI: -0.89, 4.29), $t(72)$ = 1.24, p= 0.219	
	Perspective * Training	**b = 2.80 (95% CI: 0.21, 5.39), $t(72)$ = 2.04, p= 0.045 ***	
	Perspective * Retention	b = 1.80 (95% CI: -0.79, 4.39), $t(72)$ = 1.31, p= 0.193	
	Feedback * Perspective * Training	b = -1.40 (95% CI: -5.07, 2.27), $t(72)$ = -0.72, p= 0.473	
	Feedback * Perspective * Retention	b = -0,70 (95% CI: -4.37, 2.97), $t(72)$ = -0.36, p= 0.719	
Frustration	Feedback	b = 0.50 (95% CI: -3.51, 4.51), $t(36)$ = 0.24, p= 0.812	0.86
	Perspective	b = -1.50 (95% CI: -5.51, 2.51), $t(36)$ = -0.72, p= 0.476	
	Time (Training)	b = -1.80 (95% CI: -4.91, 1.31), $t(72)$ = -1.09, p= 0.278	
	Time (Retention)	*b = -3.00 (95% CI: -6.11, 0.11), $t(72)$ = -1.82, p = 0.073 ·*	
	Feedback * Perspective	b = -2.30 (95% CI: -7.97, 3.37), $t(36)$ = -0.78, p= 0.440	
	Feedback * Training	b = 0.70 (95% CI: -3.70, 5.10), $t(72)$ = 0.30, p= 0.765	
	Feedback * Retention	b = -1.80 (95% CI: -6.20, 2.60), $t(72)$ =- 0.77, p= 0.442	
	Perspective * Training	b = 1.30 (95% CI: -3.10, 5.70), $t(72)$ = 0.56, p= 0.578	
	Perspective * Retention	b = -0.20 (95% CI: -4.60, 4.20), $t(72)$ = -0.09, p= 0.932	
	Feedback * Perspective * Training	b = -0.40 (95% CI: -6.63, 5.83), $t(72)$ = -0.12, p= 0.904	
	Feedback * Perspective * Retention	b = 3.20 (95% CI: -3.03, 9.43), $t(72)$ = 0.97, p= 0.334	
