# Supplementary figures and images for: Immersive virtual reality for learning exoskeleton-like virtual walking: a feasibility study
Source: J Neuroeng Rehabil. 2024 Nov 1;21:195. doi: 10.1186/s12984-024-01482-y (PMC11531127; doi:10.1186/s12984-024-01482-y)

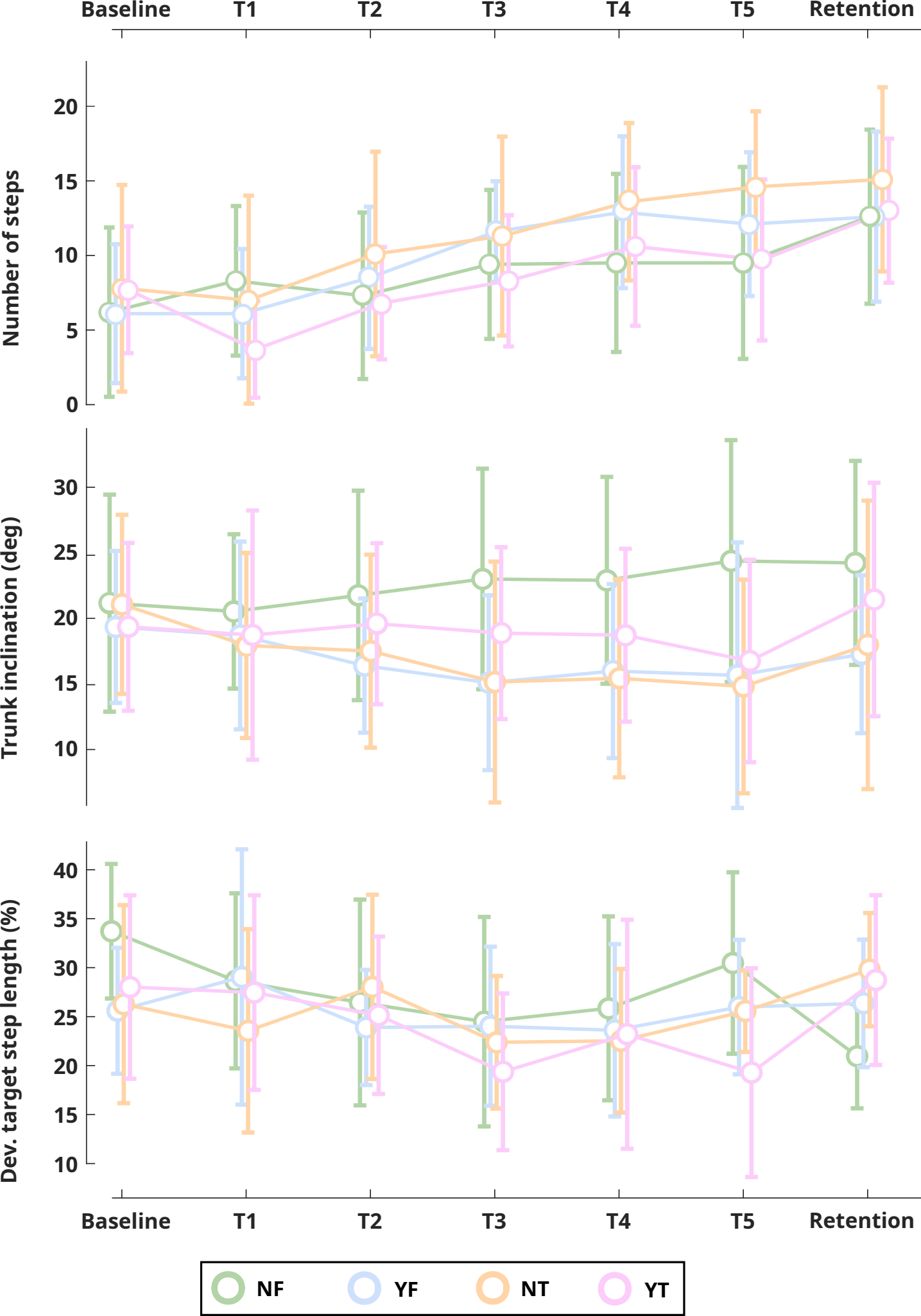

Supplement: Supplementary file 7 — Additional file 7. [file 12984_2024_1482_MOESM7_ESM.pdf]
